# Multiple Changes of Gene Expression and Function Reveal Genomic and Phenotypic Complexity in SLE-like Disease

**DOI:** 10.1371/journal.pgen.1005248

**Published:** 2015-06-09

**Authors:** Maria Wilbe, Sergey V. Kozyrev, Fabiana H. G. Farias, Hanna D. Bremer, Anna Hedlund, Gerli R. Pielberg, Eija H. Seppälä, Ulla Gustafson, Hannes Lohi, Örjan Carlborg, Göran Andersson, Helene Hansson-Hamlin, Kerstin Lindblad-Toh

**Affiliations:** 1 Department of Animal Breeding and Genetics, Swedish University of Agricultural Sciences (SLU), Uppsala, Sweden; 2 Science for Life Laboratory, Department of Medical Biochemistry and Microbiology, Uppsala University, Uppsala, Sweden; 3 Department of Clinical Sciences, Swedish University of Agricultural Sciences (SLU), Uppsala, Sweden; 4 Research Programs Unit, Molecular Neurology; Department of Veterinary Biosciences, University of Helsinki and Folkhälsan Research Center, Helsinki, Finland; 5 Department of Clinical Sciences, Division of Computational Genetics, Swedish University of Agricultural Sciences (SLU), Uppsala, Sweden; 6 Broad Institute, Cambridge, Cambridge, Massachusetts, United States of America; University of Washington, UNITED STATES

## Abstract

The complexity of clinical manifestations commonly observed in autoimmune disorders poses a major challenge to genetic studies of such diseases. Systemic lupus erythematosus (SLE) affects humans as well as other mammals, and is characterized by the presence of antinuclear antibodies (ANA) in patients’ sera and multiple disparate clinical features. Here we present evidence that particular sub-phenotypes of canine SLE-related disease, based on homogenous (ANA^H^) and speckled ANA (ANA^S^) staining pattern, and also steroid-responsive meningitis-arteritis (SRMA) are associated with different but overlapping sets of genes. In addition to association to certain MHC alleles and haplotypes, we identified 11 genes (*WFDC3*, *HOMER2*, *VRK1*, *PTPN3*, *WHAMM*, *BANK1*, *AP3B2*, *DAPP1*, *LAMTOR3*, *DDIT4L* and *PPP3CA*) located on five chromosomes that contain multiple risk haplotypes correlated with gene expression and disease sub-phenotypes in an intricate manner. Intriguingly, the association of *BANK1* with both human and canine SLE appears to lead to similar changes in gene expression levels in both species. Our results suggest that molecular definition may help unravel the mechanisms of different clinical features common between and specific to various autoimmune disease phenotypes in dogs and humans.

## Author Summary

Autoimmune disorders display complex phenotypes with clinically diverse manifestations, which together with complex genetic inheritance and environmental factors triggering the disease may complicate the diagnosis and investigation of the disease mechanism. The use of dog breeds may facilitate the analysis of genetic factors based on genetic homogeneity within a breed. We performed genetic analysis of two diseases common in dogs, immune-mediated rheumatic disease (IMRD) and steroid-responsive meningitis-arteritis (SRMA) that are similar to human SLE and a group of vasulitides such as Kawasaki disease, Henoch-Schönlein purpura and Behçet’s disease, correspondingly. We identified eleven genes along with specific alleles and genotypes for the major histocompatibility complex II involved in susceptibility, and studied their expression. The genes shared between the two diseases may be involved in the common immune signaling pathways and hence account for the common clinical signs, whereas the phenotype-specific genes may be implicated in particular pathways active in certain tissues and organs, and thereby may be responsible for characteristic manifestations seen only in one of the diseases. Further, the similarity between human and dog SLE at the genetic and functional levels demonstrated by the association of the *BANK1* gene in both species indicates the common cross-species mechanisms of autoimmunity and may help identification of novel disease genes and pathways.

## Introduction

SLE is a chronic autoimmune disorder caused by multiple genetic and environmental risk factors. The disease tends to be clinically heterogeneous [1], with manifestations ranging from relatively mild symptoms such as skin rash to severe impairment of functions of kidney, heart, lung, central nervous system and other organs [2, 3]. A hallmark of the disease is the production of autoantibodies directed to self-antigens located in the nucleus, cytoplasm or on the cell surface. Antinuclear antibodies (ANA) are found in more than 95% of human SLE cases [4].

While SLE and SLE-related diseases were first described in human patients, they are also seen in other species including dogs with similar clinical manifestations [5–8], which makes dog a good comparative model for genetic studies of human SLE. Nova Scotia duck tolling retriever (NSDTR) dogs appear to be predisposed to an SLE-like disease called immune-mediated rheumatic disease (IMRD) [5], and also show strong predisposition to another related immune-mediated disease, steroid-responsive meningitis-arteritis (SRMA), which share some features with human vasculitides including Kawasaki disease [9–14], Henoch-Schönlein purpura [15] and Behçet’s disease [16].

It was shown in the recent years that circulating autoantibodies could be linked to specific types of both canine and human autoimmune diseases [8, 17–19]. The immunofluorescent ANA test reveals two major patterns of ANA, homogeneous with a concomitant cytoplasmic and chromosomal reactivity and speckled with only cytoplasmic antigens stained. A previous study showed that among canine IMRD cases positive for indirect immunofluorescence (IIF)-ANA, 61% showed the speckled pattern (ANA^S^), whereas 39% displayed homogeneous phenotype (ANA^H^) [5]. While the link between autoantibodies and sub-phenotypes of disease may be evident, especially in the case of tissue-specific antigens, the genetic factors behind this connection are not well known.

To date, autoimmune diseases in both humans and dogs have been found associated with both major histocompatibility complex (MHC) class II alleles [20–25] and many other susceptibility genes [26, 27]. Out of 40 loci that have been associated with human SLE the causative variant and susceptibility mechanism has been described only for a few [28], leaving a lot of remaining work in understanding genome function and genotype-phenotype correlations.

Overall, dogs share many of man’s common diseases, but they also have a unique genome structure, which greatly facilitates genome wide association studies (GWAS) and, compared to human studies, significantly fewer genetic markers and samples are required for gene mapping in dogs [29, 30]. This is a result of the canine genome architecture characterized by high linkage disequilibrium within breeds being 40- to 100-fold longer compared to that observed in the human genome. The genomic architecture of domestic dogs has been formed by multiple genetic bottlenecks, founder effects and restricted breeding practices [30]. Gene mapping in dogs has proven successful with only ~100 cases and ~100 controls for complex traits and the list of disease-causing genes that have been identified in dogs is constantly growing (some are reviewed in [31, 32]).

In fact, in the first successful GWAS for a canine complex trait we mapped five loci for IMRD and SRMA using only 57 controls and 81 cases including 37 with IMRD and 44 with SRMA [27]. To replicate these loci we performed fine-mapping using a total of 160 cases including 82 dogs with IMRD and 78 with SRMA and 173 controls, and replicated all of the five loci in at least one of the phenotypes analysed [27].

In this study, we have performed further functional and genetic dissection of all five GWAS loci identified previously. We present evidence for the association of 11 genes located on five chromosomes and specific genotypes for the canine leukocyte antigen (DLA, equivalent to MHC) class II to different sub-phenotypes of SLE-related disease and SRMA in dogs, as well as study the correlation between the associated SNPs and haplotypes and an altered expression of genes in the respective loci.

## Results

### Association of the speckled ANA pattern with DLA class II risk haplotype and general homozygosity for DLA haplotypes with the homogeneous ANA phenotype

**Fig 1:**
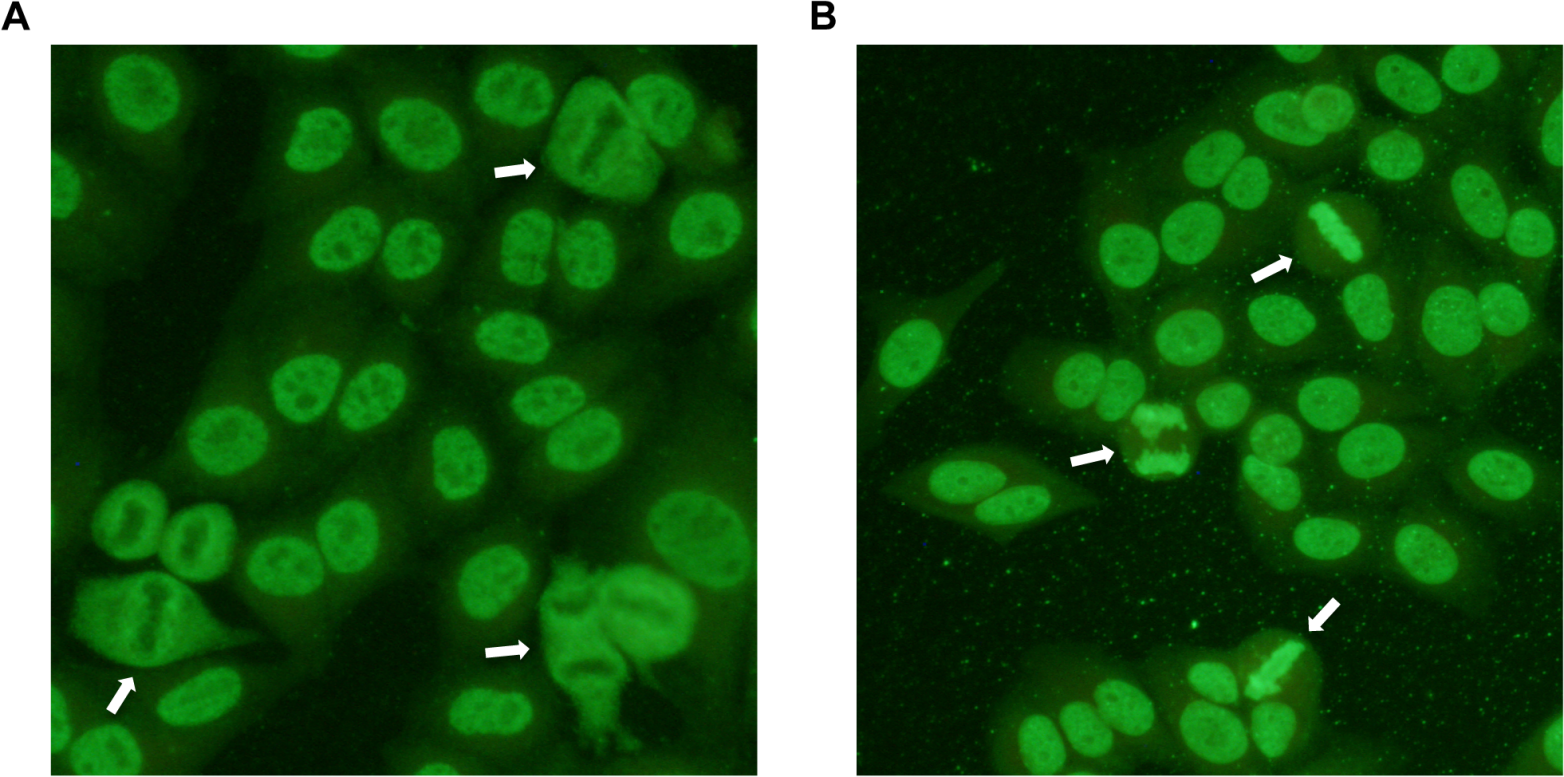
Indirect immunofluorescence staining of HEp-2 cells treated with serum from patient dogs. ANAs directed against specific nuclear antigens reveal different patterns on stained cells. **A)** Speckled ANA pattern. Arrows point at mitotic cells with negatively stained chromosomes surrounded by positive nucleosome staining. **B)** Homogeneous ANA pattern. The positive staining of chromatin in dividing cells is shown by arrows.

We first performed an indirect immunofluorescent ANA test on serum from 59 cases and 63 healthy control NSDTRs. Of these, 26 cases were classified as ANA^H^ and 27 cases as ANA^S^ (six cases could not be classified due to lack of serum) (**Fig 1**), while all healthy controls were ANA-negative. The polymorphic exon 2 was sequenced for each of the DLA-DRB1,-DQA1 and-DQB1 genes in all dogs (**S1 Table**). A total of five DLA-DRB1, four DLA-DQA1 and five DLA-DQB1 alleles, forming five different haplotypes were identified (**S2 and S3 Tables**). Ten different genotypes were observed in the study population (**Table 1**). Association analysis was performed for alleles, haplotypes and genotypes for the ANA^H^ and ANA^S^ case groups separately as well as the combined case group, and each was compared to controls (**Table 1, S2 and S3 Tables**).

**Table 1:** Genotype frequencies in the NSDTR population indicate an increased frequency for ANA^S^ dogs homozygous for haplotype 2 (DLA-DRB1*00601/DQA1*005011/DQB1*02001) compared to controls and an increase in frequency for ANA^H^ dogs with a homozygous haplotype (No 1.1 and 3.3) compared to controls.

Genotype No	Haplotype No	ANA % (n = 59)	ANA^H^ % (n = 26)	ANA^S^ % (n = 27)	Controls % (n = 63)	Total population % (n = 122)	OR	P-value
1	1.1	20.3	**38.5**	0.0	**9.5**	14.8	5.9	NA
2	2.2	40.7	7.7	**77.8**	**14.3**	27.0	21.0	<0.0001
3	3.3	10.2	**19.2**	3.7	**3.2**	6.6	7.3	NA
4	1.2	8.5	3.8	11.1	28.6	18.9	-	-
5	1.3	11.9	23.1	0.0	17.5	14.8	-	-
6	1.5	5.1	3.8	3.7	4.8	4.9	-	-
7	2.3	1.7	3.8	0.0	12.7	7.4	-	-
8	2.5	1.7	0.0	3.7	4.8	3.3	-	-
9	3.5	0.0	0.0	0.0	3.2	1.6	-	-
10	1.4	0.0	0.0	0.0	1.6	0.8	-	-

S = Speckled, H = Homogeneous

Bold indicate between what groups the largest allele frequency difference occurred and where statistics were performed (OR and P-values).

There was a significant association with haplotype 2 in ANA^S^ cases compared to the control group (OR = 9.7 and p = <0.0001) (**S3 Table**), and an even higher OR in homozygote individuals (OR = 21.0 and p<0.0001; genotype 2; 77.8% in ANA^S^ cases vs. 14.3% in controls) (**Table 1**). In total, 93% of the twenty-seven ANA^S^ dogs were either homo- or heterozygous for haplotype 2 (DLA-DRBI*00601/DQA1*005011/DQB1*02001) and twenty-one of them (77.8%) were homozygous.

No significant association was observed between the haplotypes or genotypes of DLA and the cases with ANA^H^ pattern. However, at the allelic level a significant association was identified for the DQA1*00601 (86.5% in ANA^H^ cases compared to 55.6% in controls; OR = 5.1 and p = 0.00017, **S2 Table**).

As homozygosity has been hypothesized to be a risk factor in itself, we removed the ANA^S^ risk genotype and analyzed the remaining data for association to homozygosity regardless of haplotype. We found an increase in homozygosity in ANA^H^ cases (62.5%) vs. controls (14.8%), implicating a general homozygous disadvantage at DLA class II for ANA^H^ dogs (OR = 9.6, p<0.0001; **S4 Table**).

### Distinct risk loci additionally contribute to susceptibility to IMRD and SRMA

**Fig 2:**
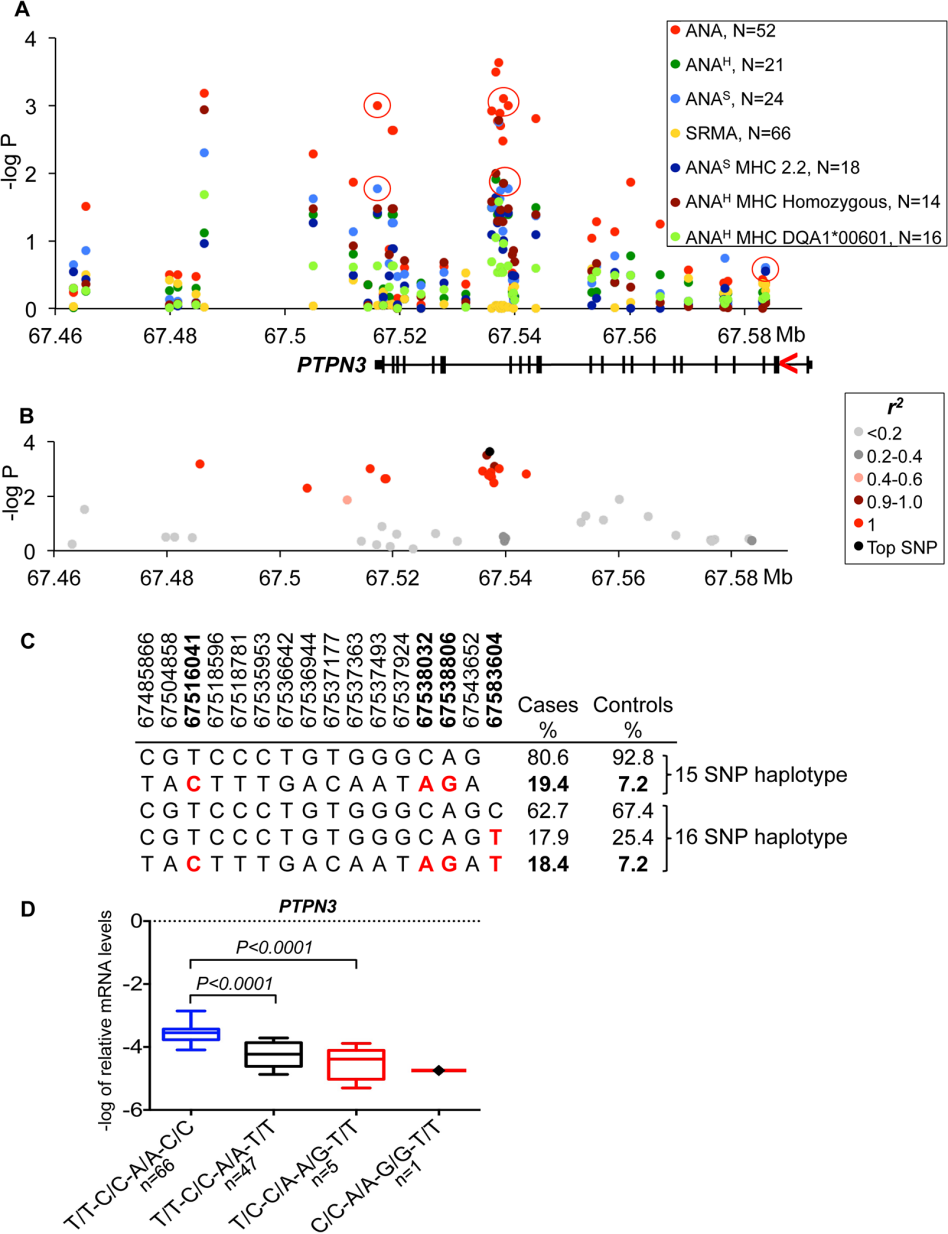
Genetic analysis of chromosome 11 locus and *PTPN3* expression. **(A)** The strongest association signal was observed for the ANA-positive IMRD phenotype and overlaps with the *PTPN3* gene. The red circles show the SNPs that correlate with gene expression and indicated for all associated sub-phenotypes. The gene structure is shown below with exons as vertical bars, the direction of transcription is indicated by red arrowhead. **(B)** A 15 SNP risk haplotype identified for ANA-positive cases, all SNPs in almost complete LD (r^2^ >0.9 for all pairs) with the top associated variant (11:67537177). **(C)** Haplotype frequencies in cases and controls. The SNPs used for expression studies are shown in bold and their risk alleles in red. **(D)** The log-transformed mRNA levels of *PTPN3* in the PBMCs of dogs with different haplotypes comprised of SNPs in the 3’-UTR and intron 18 and two synonymous SNPs in exons 18 and 3. The protective haplotype is T/T-C/C-A/A-C/C is shown in blue color, the associated risk haplotypes T/C-C/A-A/G-T/T and C/C-A/A-G/G-T/T—in red color. The *PTPN3* gene is down-regulated 7-fold in the heterozygous risk haplotype compared to the protective haplotype. Boxes represent interquartile range 25–75% with median, and 5–95 percentile range with maximum and minimum values. The dog number in each group is shown next to the haplotypes. The gene expression was normalized to the levels of the reference gene *TBP* and analyzed using a one-way ANOVA. All phenotypes and SNP labels as well as gene structures, expression and normalization presented here are unified with the figures for other loci.

**Fig 3:**
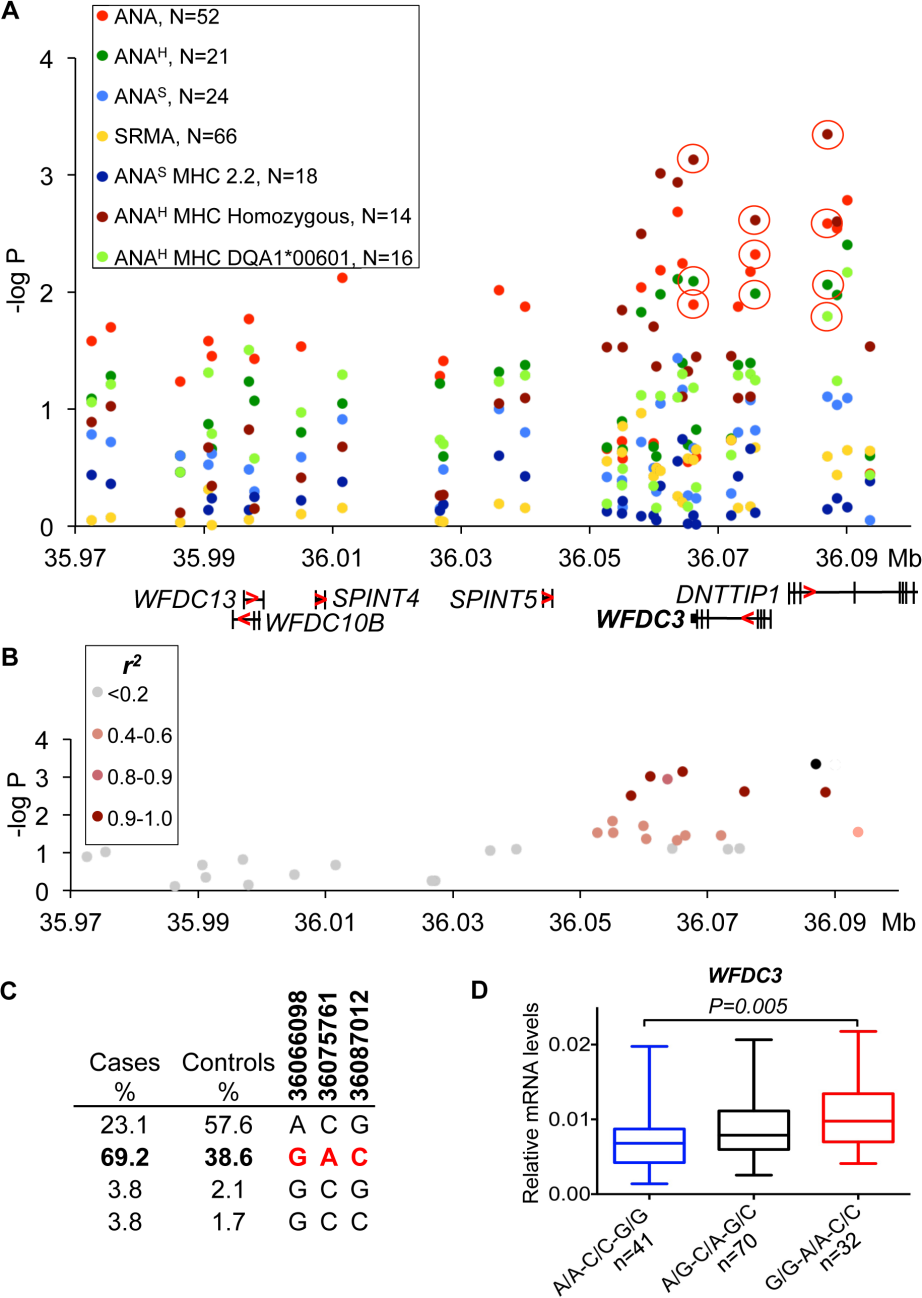
Genetic and gene expression analyses for chromosome 24 locus. **(A)** The strongest association was observed for the ANA^H^ dogs with a homozygous DLA haplotype (14 patient dogs) in the region that contains *WFDC3* and *DNTTIP1* genes. Differentially expressed gene *WFDC3* is labeled with bold font. **(B)** r^2^ analysis was performed on the top SNP (24:36087012) and revealed a seven SNP risk haplotype (r^2^>0.9) overlapping the *WFDC3* and *DNTTIP1* genes. **(C)** Haplotype frequencies in cases and controls. **(D)** The increased transcript level of *WFDC3* in the risk haplotype made of two synonymous SNPs (24:36066098, 24:36075761) and the top SLE variant.

**Fig 4:**
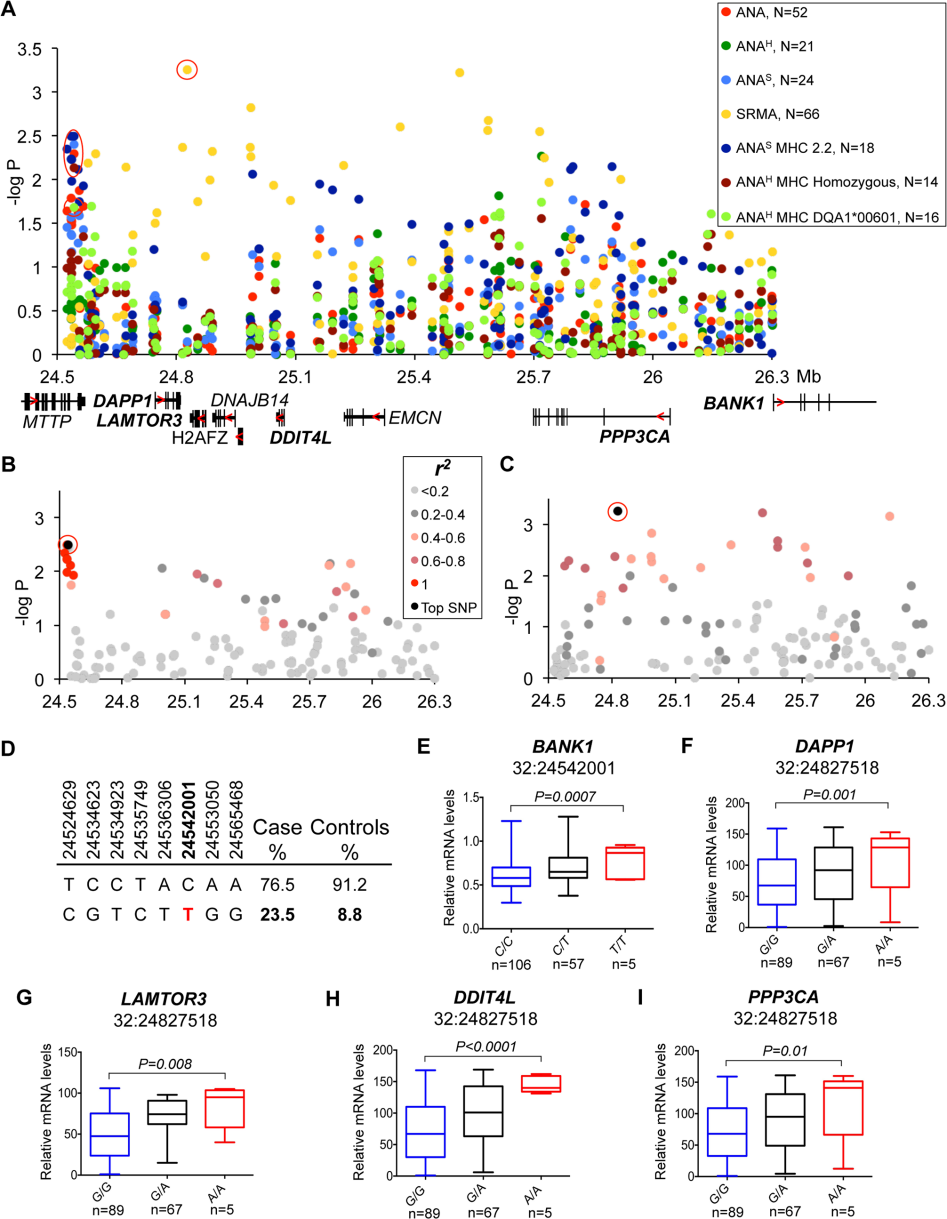
Genetic and gene expression analyses for chromosome 32 locus. **(A)** Strong association to the locus was observed in ANA and independently in SRMA-affected dogs. The signal for ANA dogs is located between the *MTTP* and *DAPP1* gene. SRMA dogs show multiple strong signals spread over a 1.3 Mb region. **(B)** ANA^S^ dogs homozygous for DLA haplotype 2 show a narrow eight SNP risk haplotype with complete LD (r^2^ = 1) with the top SNP (32:24542001). **(C)** No strong LD was identified for the top SNP (32:24827518) in SRMA dogs, but r^2^ of 0.6–0.8 occurs throughout the region. **(D)** Haplotype frequencies in cases and controls in ANA^S^ dogs. **(E-I)** Genes with differential expression in the blood cells associated with two top variants (32:24542001 for ANA^S^-DLA 2.2) with *BANK1* **(E)**, and (32:24827518 for SRMA) with *DAPP1* **(F)**, *LAMTOR3* **(G)**, *DDIT4L* **(H)**, *PPP3CA* **(I)**.

**Fig 5:**
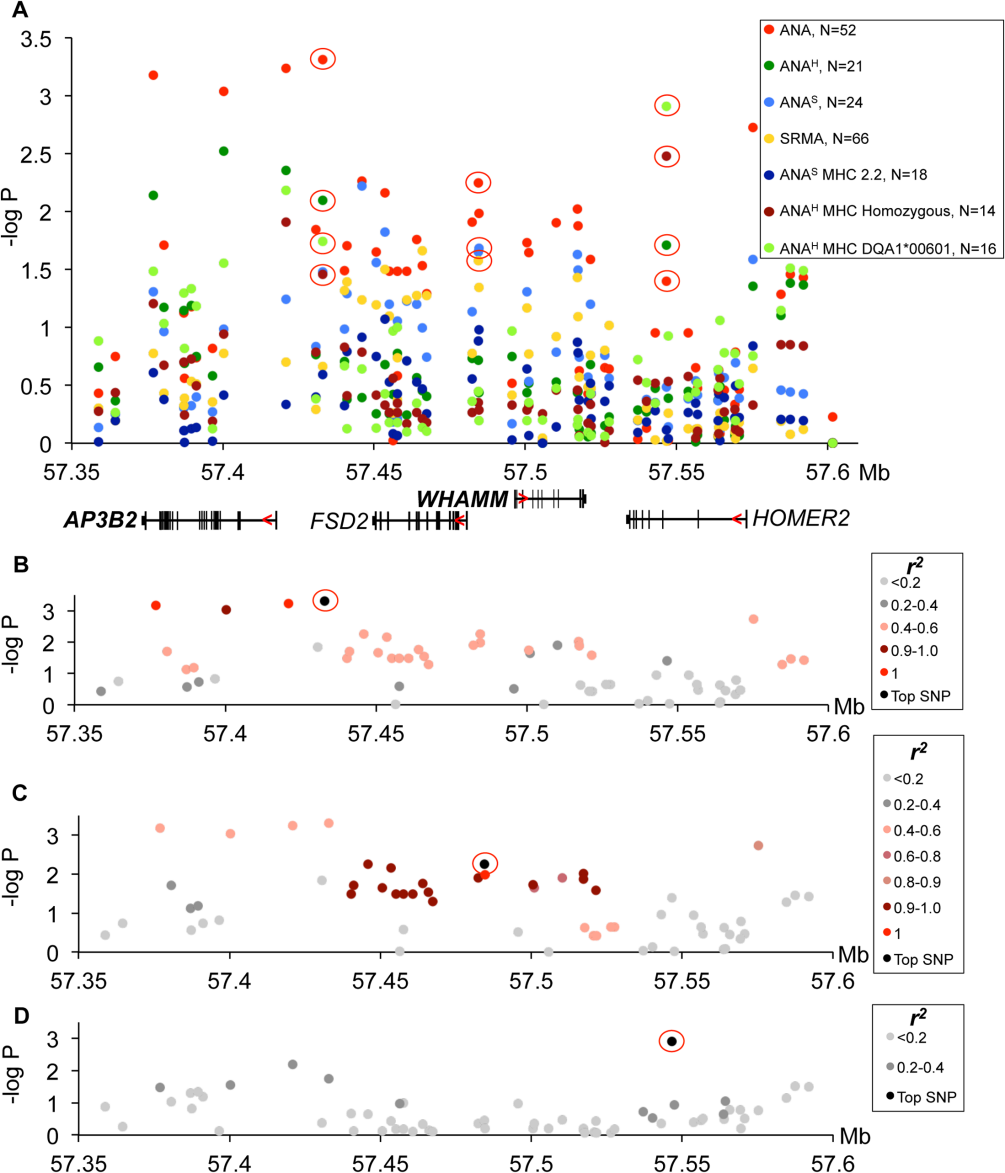
Genetic analysis of chromosome 3 locus. **(A)** The strongest association signals were observed in all ANA-positive dogs in the *AP3B2* and in the intergenic region between *AP3B2* and *FSD2* genes, followed by a single SNP peak in the *HOMER2* gene (3:57546568) associated ANA^H^ dogs with the risk allele DLA-DQA1*00601. **(B)** The top SNP for ANA-positive dogs (3:57432981) occurs in a four SNP haplotype with strong LD (r^2^>0.9). **(C)** Another associated region was identified for ANA-positive dogs (top SNP 3:57484486) in an 18 SNP haplotype with strong LD (r^2^>0.9). **(D)** ANA^H^ dogs with the risk DLA-DQA1*00601 show one top SNP (3:57546568) independent of other variants.

**Fig 6:**
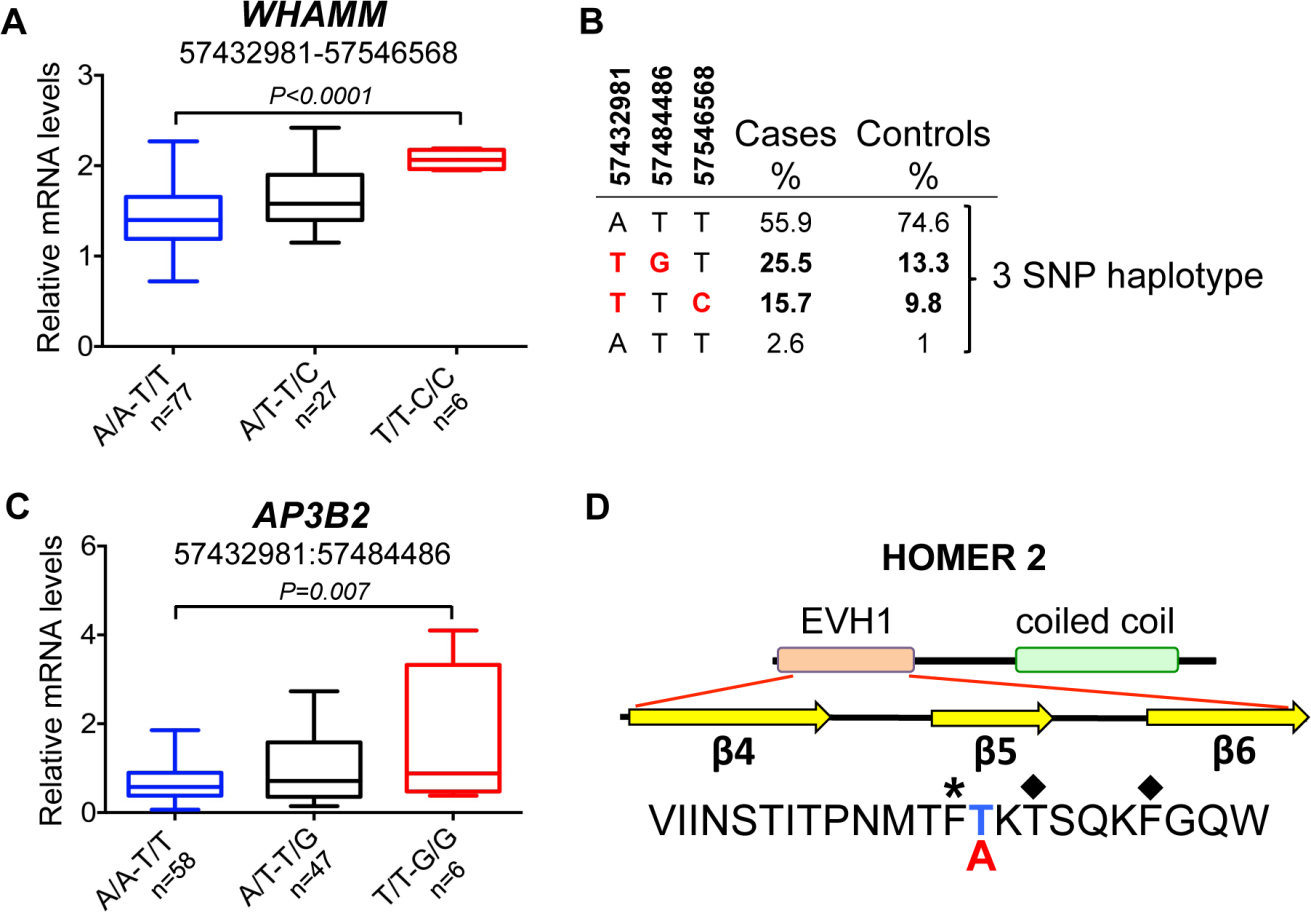
Chromosome 3 risk haplotypes and their effects on genes. **(A)** The haplotype made of SNPs 3:57432981–3:57546568 is best associated with expression changes of *WHAMM* (1.5-fold up-regulation in the risk, *P<0*.*0001*), while *AP3B2* was not altered in this haplotype. **(B)** The frequencies of the haplotypes made of the top SNPs that represent three independently associated regions. **(C)** The second risk haplotype (3:57432981–3:57484486) is stronger associated with up-regulation of *AP3B2* (1.5-fold, *P = 0*.*007*) and to a lesser extent with *WHAMM* (1.2-fold up-regulation, *P = 0*.*016*, **S4 Fig**). **(D)** The schematic structure of the HOMER2 protein with the EVH1 domain and a coiled coil region. The amino acid sequence is shown below for the region from β4 to β6 strands. The nonsynonymous variant 3:57546568 changes Thr (blue color, protective) to Ala (red color, risk) in the HOMER2 protein. An asterisk marks the hydrophobic core residue and the two amino acids critical for the peptide binding site are marked with diamonds.

**Fig 7:**
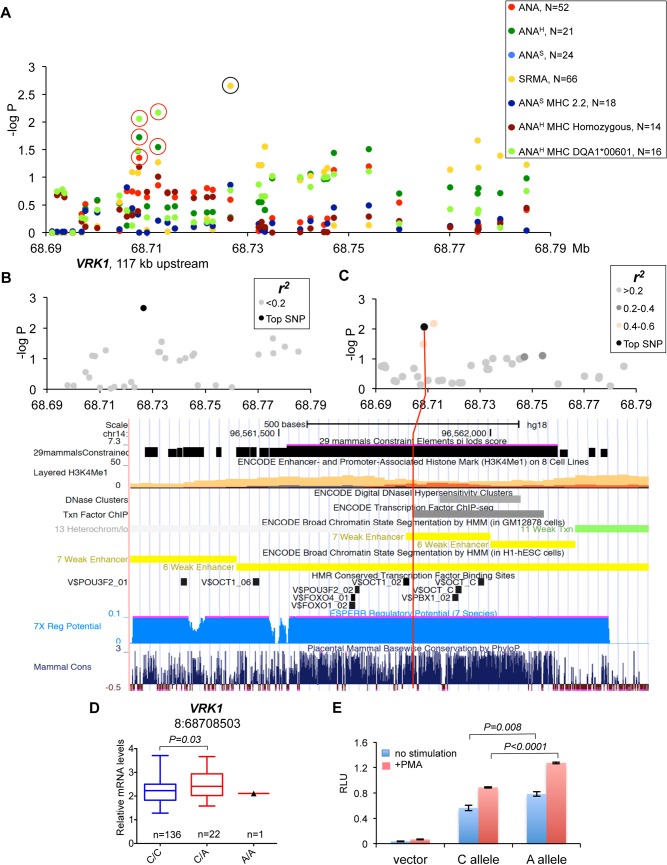
Genetic and gene expression analyses for chromosome 8 locus. **(A)** Two independent strong association signals were observed for SRMA affected dogs (8:68726546) and ANA^H^ dogs with DLA risk allele DLA-DQA1*00601(8:68712185 and 8:68708503). The only closest gene, *VRK1*, is located over 100 kb upstream of the associated region while there are no protein-coding genes for more than 1.9 Mb downstream. The black circle indicates the SRMA SNP not associated with expression changes of *VRK1*, the red circles—SNPs associated with *VRK1* expression. **(B)** No SNPs were in LD with the top SRMA SNP. **(C)** The SNP 8:68708503 for ANA^H^ dogs with DLA risk allele DLA-DQA1*00601 is not in LD with any other genotyped variants, but have a strong regulatory potential. The 1 kb region aligned with the corresponding human fragment is shown below with several tracks for gene regulation and conservation displayed. **(D)** Expression levels of the *VRK1* gene in the blood cells of healthy NSDTRs stratified by SNP 8:68708503. Only one dog homozygous for the risk A allele was available. **(E)** The DNA fragment with the SNP 8:68708503 was cloned in the luciferase reporter vector and following transfection in K562 cells the protein lysate was assayed for enzyme activity. The risk allele A enhances luciferase expression comparing to the protective C allele in both unstimulated and stimulated with PMA cells.

To search for candidate variants, the five genetic risk loci on CFA 3, 8, 11, 24 and 32 that were previously identified by GWAS to be associated to IMRD and SRMA [27] were re-sequenced in four ANA-positive cases, two SRMA cases and three healthy dogs using Nimblegen capture and Illumina sequencing. Using standard methods, a total of 13,084 SNPs and 2,780 indels were detected. No structural changes or CNVs that differed between cases and controls were identified. Among those, 426 SNPs and 88 indels showed a potential functional effect by SeqScoring [33]. Next, 308 SNPs following the risk haplotype patterns were chosen for genotyping in the entire sample set (**S5 Table**). For each locus, association analysis was performed between the 132 healthy controls and each of the different sub-phenotypes: 1) SRMA-affected dogs (N = 66), 2) all ANA-affected dogs (N = 52), 3) ANA^S^ (N = 24) and 4) ANA^H^ staining pattern (N = 21). Furthermore, two conditional analyses were performed where only ANA^S^ dogs homozygous for DLA risk haplotype 2 (N = 18) and ANA^H^ dogs homozygous for DLA (N = 14) and ANA^H^ dogs with the DQA1*00601 allele (N = 16) were included respectively. For the risk locus on chromosome 11 the strongest association was observed with all ANA dogs (**Fig 2**), for the risk locus on chromosome 24 the strongest associated sub-phenotype was the ANA^H^ dogs homozygous for DLA (**Fig 3**), while the risk locus on chromosome 32 showed two independent association signals for SRMA and ANA^S^ with and without DLA association (**Fig 4);** and the risk locus on chromosome 3 showed signals for all ANA, and for ANA^H^ and ANA^S^ tagged by different haplotypes, and to a lesser extent to SRMA (**Figs 5 and 6**). Finally, the risk locus on chromosome 8 also showed two separate signals to SRMA and ANA^H^ dogs associated DLA (**Fig 7**). Of note, in the current report, as we further investigate the already associated and replicated regions [27], we used raw p-values focusing on the highest peaks for each sub-phenotype to investigate its effect on gene expression. These regions have already been replicated in our previous genome-wide association study with *P* values ranging between 10^-5^-10^-6^, with three loci on chromosomes 3, 11 and 24 reaching genome-wide significance following fine-mapping and validation with *P* values of 10^-11^-10^-13^ [27]. The results are discussed in more detail below, together with expression analysis of the genes at each locus.

#### Down-regulation of *PTPN3* is associated with risk for ANA-positive IMRD

On chromosome 11, the strongest association was seen between all ANA cases and a SNP (CanFam2.0 chr11:67,537,177, p = 2.3*10^–4^) located within the non-receptor type 3 protein tyrosine phosphatase gene (*PTPN3)* (**Fig 2A**). The top SNP was in almost complete LD with 14 other SNPs (r^2^ >0.9 for all pairs) (**Fig 2B**) forming a 58 kb haplotype overlapping the 3’ end of the gene. The risk haplotype had an allele frequency of 19.4% in cases versus 7.2% in controls (**Fig 2C**). Based on the strong LD, three SNPs from the haplotype were selected (one in the 3’UTR, a SNP in intron 18 and one synonymous SNP in exon 18: 11:67,516,041, 11:67,538,032 and 11:67,538,806, respectively) and then the genotypes were correlated with *PTPN3* mRNA levels measured in total RNA purified from peripheral blood mononuclear cells (PBMC) of 167 healthy NSDTRs (**Fig 2D**). The expression of *PTPN3* was substantially down regulated in heterozygotes (7-fold change, *P*_*ANOVA*_ *<0*.*0001*). As only one dog was found to be homozygous for the risk haplotype among the 167 healthy dogs, it was not included in the statistical analysis, but we noted that this dog showed extremely low levels of *PTPN3* expression. To examine whether all three genotypes could be represented in the expression analysis, a synonymous SNP in exon 3 (11:67,583,604, p = 0.43) falling just outside the most associated haplotype (r^2^ = 0.23) was selected for analysis in combination with the other three SNPs from the risk haplotype. This SNP contributed to the down-regulation both independently and in combination with the risk haplotype (**Fig 2D**), suggesting a cumulative effect on gene expression levels from the risk haplotype and the exon 3 variant. Among the cases used in the association study, only four dogs were homozygous for the risk haplotype C/C-A/A-G/G-T/T (11:67,516,041, 11:67,538,032, 11:67,538,806 and 11:67,583,604, respectively) and three of them showed severe IMRD that eventually led to death (one dog died at the age of 19 months, and two at 6 years of age).

#### Up-regulation of *WFDC3* correlates with risk for ANA^H^ in combination with homozygosity for DLA

The strongest association for the risk locus on chromosome 24 was identified for a sub-phenotype of ANA^H^ in combination with a homozygous DLA haplotype (**Fig 3A**). Seven SNPs in high LD (r^2^>0.9) (**Fig 3B**) define a risk haplotype that overlaps two genes coding for WAP four-disulfide core domain protein 3 (*WFDC3*) and terminal deoxynucleotidyltransferase interacting protein 1 (*DNTTIP1*) and is present at a frequency of 69.2% in cases and 38.6% in controls (**Fig 3C**). The mRNA expression in PBMCs of these two genes was analyzed in detail. Several other genes located upstream in the locus *WFDC13*, *WFDC10B*, *SPINT4* and *SPINT5* were found not to be expressed in either PBMCs or 13 diverse dog tissues analyzed. The top SNP, located in intron 3 of *DNTTIP1* (24:36,087,012, p = 4.5*10^–4^), is associated with expression of *WFDC3* (1.4-fold change, *P*_*ANOVA*_ *= 0*.*0075*) (**S6 Table**), whereas neither alternative splicing nor expression changes for the *DNTTIP1* gene were detected. Both genes are separated by less than 3 kb and placed head-to-head, whereby intronic SNPs in *DNTTIP1* could serve as distal upstream enhancers for *WFDC3*. The two synonymous variants in exon 3 and exon 5 of *WFDC3* are also strongly associated with gene expression (24:36,075,761, 1.4-fold, *P*_*ANOVA*_ *= 0*.*0039*, r^2^>0.9 with 24:36,087,012; 24:36,066,098, 1.4-fold, *P*_*ANOVA*_ *= 0*.*0076*, r^2^>0.9) (**S6 Table**). Since all three variants are in high LD, and the two synonymous SNPs have stronger or equal association with transcription compared with the top SLE SNP 24:36,087,012, we decided to determine the combined effect of the three-SNP haplotype (24:36,066,098, 24:36,075,761, 24:36,087,012) on gene expression. The *WFDC3* gene is upregulated 1.4-times in the risk haplotype G/G-A/A-C/C (*P*_*ANOVA*_ *= 0*.*005*) compared to protective A/A-C/C-G/G (**Fig 3D**).

#### Two independent association signals on chromosome 32 link to ANA and SRMA respectively

Association for the risk locus on chromosome 32 was observed in multiple sub-phenotypes. In ANA^S^ dogs that were homozygous for DLA haplotype 2 and to a lesser extent in “all ANA” and homogeneous ANA with DLA DQA1*00601 allele and general homozygosity for DLA (**Fig 4A**), the signal was located between the *MTTP* and *DAPP1* genes and included eight SNPs in complete LD (r^2^ = 1) on a 41 kb haplotype (32:24,514,629–24,565,468, best association: p = 4.0*10^–4^) (**Fig 4B**). In contrast to ANA phenotypes, the association with SRMA did not form a single risk-haplotype, but instead was spread over a 1.3 Mb region (32:24,827,518–32:26,115,349) (**Fig 4A**). While no strong LD was identified, 11 SNPs out of 131 genotyped for this chromosomal region have an r^2^ between 0.6–0.8 across the whole region (**Fig 4C**).

To examine the functional effect of the risk genotypes, we measured mRNA expression of all nine genes from the locus in the PBMCs of healthy dogs (**Fig 4E–4I, S1 Fig**). We genotyped 24 highly associated SNPs across the entire region and correlated them with expression of the genes (**S7 Table**). Interestingly, the ANA^S^/ANA/ANA^H^ associated haplotype that occurs more frequently in patients (23.5%) than healthy controls (8.8%) (**Fig 4D**) was only significantly correlated with a 1.5-fold up-regulation of the *BANK1* gene (*P*_*ANOVA*_ *= 0*.*0007*), located 1.5 Mb downstream from the top SNP 32:24,542,001, which suggests a long-range regulatory effect (**Fig 4E**).

The top SNP (chr32:24,827,518) for SRMA, on the other hand, was also the SNP associated with the most significant expression changes for the following genes: *DAPP1*, *LAMTOR3*, *DDIT4L* and *PPP3CA*. The risk allele A correlated with enhanced gene expression levels (1.7-fold for *DAPP1*, *P*_*ANOVA*_ *= 0*.*001*; 1.9-fold for *LAMTOR3*, *P*_*ANOVA*_ *= 0*.*008*; 2.5-fold for *DDIT4L*, *P*_*ANOVA*_ *<0*.*0001*; 2-fold for *PPP3CA*, *P*_*ANOVA*_ *= 0*.*01*) (**Fig 4F–4I**).

#### Complex changes of gene functions in the chromosome 3 locus are associated with both ANA-positivity and SRMA

Association of the risk locus on chromosome 3 was identified for ‘all ANA’ dogs including speckled and homogeneous sub-phenotypes (**Fig 5A**) with the top SNP (chr3:57,432,981, p = 4.9*10^–4^) linked to a four SNP haplotype in strong LD (r^2^>0.9) (**Fig 5B**), where the haplotype overlaps the *AP3B2* gene and extends towards the *FSD2* gene. A second haplotype consisting of 18 SNPs in strong LD (r^2^>0.9) with a SNP at chr3:57,484,486 (**Fig 5C**) was located upstream of the *WHAMM* gene and associated to the ‘all ANA’ (p = 5.6*10^–3^) and the speckled phenotypes (p = 2.2*10^–2^). Interestingly, this complex haplotype was associated with SRMA (p = 2.6*10^–2^) (**Fig 5A**). Separately, we also identified an association for the phenotype of ANA^H^ + DLA-DQA1*00601 to a SNP located in the *HOMER2* gene (chr3:57,546,568, p = 1.2*10^–3^, **Fig 5A**). No LD was observed between this SNP and the other strongly associated SNPs (**Fig 5D**).

Next, the mRNA expression of the four genes located within the borders of the associated region was analyzed. We found that three genes, *AP3B2*, *WHAMM* and *HOMER2*, are ubiquitously expressed while *FSD2* expression is restricted to skeletal muscle, heart, kidney, testis, skin, and very low levels detected in cartilage (**S2 Fig**). Due to low minor allele frequencies, we could not collect enough tissue samples from genetically different NSDTRs to analyze *FSD2* expression, and thus we could not rule out entirely the possibility for this gene to be affected by the associated variants in a particular tissue.

However, the most associated SNP for ‘all ANA’ (located between *AP3B* and *FSD2)* was most associated with expression of *WHAMM*. The minor risk allele T correlates with up-regulation of *WHAMM* mRNA expression (1.4-fold, *P*_*ANOVA*_<0.0001), while showing almost no biologically significant effect on *AP3B2* (1.07-fold median change, *P = 0*.*036*) (**S8 Table**). On the contrary, the associated SNP 3:57,484,486 located upstream of *WHAMM*, exerted a more profound effect on *AP3B2* transcription (1.5-fold, *P*_*ANOVA*_ = 0.004), thus indicating the existence of negative cross-regulation of the two genes.

The expression of *HOMER2* was not associated with any of the SLE-related variants, although differential regulation of the gene marginally associated with some genetic variants was found (**S3A Fig**), suggesting that there may be an independent eQTL variant(s) not directly related to the sub-phenotypes studied here. When a 2 SNPs haplotype (3:57,432,981–3:57,546,568) was considered by combining the associated SNPs at the two ends of this locus (near *AP3B2* and within *HOMER2*), the effect on *WHAMM* increased compared to when either of the two SNPs were examined separately (1.5-fold, *P*_*ANOVA*_<0.0001, **Fig 6A, S3B Fig**).

Interestingly, the top SNP 3:57,432,981 is a common variant tagging two risk sub-haplotypes: (3:57,432,981–3:57,484,486) and (3:57,432,981–3:57,546,568) occurring in 26% and 16% of patients, correspondingly (**Fig 6B**). While the first haplotype is associated more with general ANA positivity, the second one is associated with homogeneous ANA staining and even more specifically with a particular DLA risk allele. This could suggest that depending on the second SNP in the risk haplotype, the combination of affected genes (*AP3B2* and to a lesser extent *WHAMM* **(Fig 6C, S4 Fig)**, or *WHAMM* and *HOMER2* (**Fig 6A and 6D**)) may determine what pathways are under impact and hence, what disease phenotype could be expected.

In contrast to all the regulatory variants seen here, the associated SNP located within *HOMER2* (chr3:57,546,568) (**Fig 5A and 5D**), is a non-synonymous variant in exon 3 of the *HOMER2* gene causing a Thr to Ala substitution. The substitution is located in the β5 strand of a highly conserved EVH1 protein domain and is close to the key amino acids participating in the formation of the HOMER2 ligand-binding site (**Fig 6D, S5 Fig**) [34, 35]. Analysis with SIFT [36] and PROVEAN [37] suggests that the variation is rather deleterious for protein function.

#### Chromosome 8 SNPs in gene desert associated with ANA^H^ and SRMA phenotypes

The association signals on chromosome 8 fall into a 2 Mb gene-desert, downstream of the *VRK1* gene. The strongest association was observed in SRMA dogs (chr8:68,726,546, p = 2.2*10^–3^), but also the phenotype: ANA^H^ + risk allele DLA-DQA1*00601 showed significant association to two SNPs (chr8:68,712,185, p = 6.7*10^–3^ and chr8:68,708,503, p = 8.7*10^–3^) (**Fig 7A**). All three variants are not in LD with each other (**Fig 7B and 7C**, **S6 Fig**). The only close gene, *VRK1*, is located over 100 kb upstream of the signals, and its expression measured in the blood cells was found associated with the two ANA^H^ risk variants when analyzed using the two available genotypes only: major protective and heterozygous (**Fig 7D**). Due to low minor allele frequencies, only one dog homozygous for the ANA^H^ risk variants was found in the study cohort, thus limiting the power of statistical analysis of the *VRK1* expression.

Among the three top variants, only SNP chr8:68,708,503 lies in a region with high regulatory potential as predicted by ESPERR [38] (**Fig 7C**). This region also contains enhancer-associated H3K4Me1 histone-modification marks in lymphablastoid cells, ENCODE ChIP-seq and DNAse I hypersensitive sites. The SNP is located only 5 bp from a highly conserved Pou5f1/Oct4 binding site [39]. In addition, the risk allele A creates a binding site for signal transducer and activator of transcription (STAT) family transcription factors. Interestingly, Oct-1 and STAT5, members of the Pou domain-containing and STAT family transcription factors, correspondingly, were shown to form stable transcription complexes upon cell activation with cytokines and induce cyclin D1 expression [40]. In order to verify the regulatory effect of this variant, we cloned the 550 bp DNA fragment in the pGL4.26 vector, and after transfection into K562 cells performed the luciferase assay. We confirmed that the risk allele A indeed enhances expression in both non-stimulated and stimulated cells (**Fig 7E**). We conclude that the SNP chr8:68,708,503 not only has a proven regulatory potential but might be involved as a part of an enhancer in the upregulation of the *VRK1* gene in the risk for ANA^H^.

## Discussion

SLE and other autoimmune diseases occurring in humans have been intensely studied over the past years due to high heritability of such diseases and the availability of modern genetic tools. The most recent review article reports over 40 loci associated with human SLE [28]. The heterogeneity of SLE reflected by the 11 diagnostic criteria established by the American College of Rheumatology (ACR) [2, 3] supports the current understanding that the genetic factors underlying such disparate clinical manifestations could be different as well. Recently, differential genetic associations with SLE based on the anti-dsDNA autoantibody status, either anti-dsDNA positive or anti-dsDNA-negative SLE, were reported [41]. The study of the relationships between the SLE risk alleles and clinical sub-phenotypes led to the finding that certain lupus manifestations are more dependent on the presence of multiple risk alleles, while others are more strongly associated with a single variant, for example, renal disease more significantly associated with HLA-DRB1, and arthritis with the protective allele of *ITGAM* [42]. Interestingly, a third group of sub-phenotypes (malar rash, discoid rash, photosensitivity, serositis, and neurological disorders) was found not to be associated with the currently known SLE susceptibility genes, suggesting either the presence of not yet identified factors or potentially non-genetic factors, such as environmental conditions or epigenetic effects.

Dogs, like other mammalian species also develop autoimmune disorders. Furthermore, the existence of canine breeds such as NSDTRs, which are predisposed to the development of autoimmune diseases, make them potentially useful for mapping disease genes and finding novel disease pathways [31]. A hallmark of SLE-related immune-mediated rheumatic disease (IMRD) in dogs is the presence of ANA autoantibodies, which display two major patterns when stained with indirect immunofluorescence, homogeneous and speckled ANA. Both sub-phenotypes have overlapping clinical and pathological features in NSDTRs such as musculoskeletal signs, including stiffness and joint pain without joint swelling, sometimes muscle pain and lymphopenia; and all dogs showed good response to corticosteroid treatment [5]. The possible link between different ANA patterns and various clinical signs for SLE was previously observed for a group of dogs including German Shepherds, NSDTRs, and several other breeds [8]. Of note, in our NSDTR sample set, skin lesions were present in the ANA^H^ group, while muscle pain and fever were slightly more frequent in the ANA^S^ group (**S9 Table**). The onset of disease in the study cohort was at a median age of 3 years for ANA^H^ dogs and less than 2 years for ANA^S^ dogs.

The MHC region has the strongest association to many autoimmune diseases in humans due to its utmost importance in the recognition of antigens; and it was also shown to be important for canine SLE [25]. Moreover, certain genotypes such as the HLA-DRB1*03:01 was found recently to be significantly associated with specific sub-phenotypes with anti-Ro/SSA and anti-La/SSB autoantibodies [43]. In dogs, we observed that different DLA genotypes and alleles were associated with either ANA^H^ or ANA^S^, which may indicate the reactivity towards certain autoantigens produced more frequently in a particular ANA pattern.

Among the seven genes associated with “all ANA”, *WFDC3*, *HOMER2* and *VRK1* are clearly not associated with ANA^S^ reactivity (**Table 2**). Moreover, for these genes the cumulative association with DLA, either general homozygosity for any DLA haplotype or the DQA1*00601 allele, plays a somewhat stronger role. The *AP3B2* gene is associated with “all ANA”; and also has equally strong signals for two phenotypes, the speckled ANA group and SRMA, but no association with ANA^H^. Interestingly, the observed genetic segregation may favor a previously suggested hypothesis based on the differences in clinical manifestations that ANA^S^ pattern could represent another SLE-related disorder, while the ANA^H^ is more similar to human SLE [5]. The *PTPN3*, *WHAMM* and *BANK1* genes are associated with both ANA sub-phenotypes and thus could be considered as common genes.

**Table 2:** Genes associated with IMRD and SRMA phenotypes.

chromosome	SNP ID	gene/phenotype	gene effect	all ANA	ANA^S^	ANA^S^+DLA	ANA^H^	ANA^H^+DLA	SRMA
11	11:67537177	*PTPN3*	down	**+**	+	+	+	+	-
24	24:36087012	*WFDC3*	up	+	-	-	+	**+**	-
32	32:24542001	*BANK1*	up	+	+	**+**	-	+	-
	32:24827518	*DAPP1*	up	-	-	-	-	-	**+**
	32:24827518	*LAMTOR3*	up	-	-	-	-	-	**+**
	32:24827518	*DDIT4L*	up	-	-	-	-	-	**+**
	32:24827518	*PPP3CA*	up	-	-	-	-	-	**+**
3	3:57484486	*AP3B2*	up	**+**	+	-	-	-	+
	3:57432981	*WHAMM*	up	**+**	+	-	+	+	-
	3:57546568	*HOMER2*	nsSNP (Thr->Ala)^1^	+	-	-	+	**+**	-
8	8:68708503	*VRK1* ^2^	up	+	-	-	+	**+**	-

The strongest genetic association to a phenotype marked with bold “**+**”, regular “+” means the gene is associated with a particular phenotype. ^1^-we observed also a trend towards down-regulation of *HOMER2* in the risk haplotype, although it did not reach statistical significance due to small sample size. ^2^-the strong genetic association signal with SRMA on chromosome 8 was not associated with *VRK1* expression levels

A function of *WHAMM* (WAS protein homolog associated with actin, Golgi membranes and microtubules), a gene previously reported to participate in Golgi transport and membrane remodeling, and cytoskeleton formation by binding to microtubules and promoting actin polymerization [44], has not been described before in immunity, while the other two common genes *PTPN3* and *BANK1* implicate major perturbations in both T and B cells. Human protein tyrosine phosphatase PTPH1 encoded by the *PTPN3* gene inhibits T cell-activation by dephosphorylating the immune tyrosine-based activation motifs (ITAM) in the TCRζ chain that results in a downstream inhibition of NF-AT [45, 46]. The observed substantial reduction of the *PTPN3* mRNA levels in dogs carrying the risk haplotypes may cause a sustained activation of TCR signaling and lead to development of autoimmune disease. The *BANK1* gene encoding the B-cell scaffold protein with ankyrin repeats was previously found associated with human SLE and other autoimmune diseases in distinct populations and ethnic groups [47–53]. The expression of the human *BANK1* gene, similarly to what we found in IMRD dogs, is up-regulated in patients carrying the risk alleles [54]. This may suggest a common disease mechanism in the human and dog diseases.

Whether or not and to what extent, the individual genes contribute to a particular ANA-staining pattern, and more generally, to specific clinical manifestations, and what interplay could be between the associated genes and their pathways, remains to be further studied. Also, while the identity of major autoantibodies present in the serum of human individuals with different ANA reactivity is already known [18], it needs to be studied in more detail in dogs.

SRMA, on the other hand, differs from IMRD by displaying predominantly neurological signs including pain, cervical rigidity, pyrexia and a polymorphonuclear pleocytosis of the cerebrospinal fluid (CSF) [10]. The disease usually occurs at a young age (4–19 months) and is characterized by inflammation of leptomeninges and vasculitis of the leptomeningeal and mediastinal blood vessels, including arteritis of heart, thymus, and also vessels of the thyroid glands and muscles [55, 56]. The disease can be treated with immunosuppressive doses of corticosteroids similarly to IMRD [14]. While the etiology is largely unknown, it has been proven that inflammatory processes in the CNS are not caused by viral or bacterial infection [14].

The strongest genetic association with SRMA detected in NSDTRs is located on chromosome 32, followed by signals on chromosome 8 and 3. Although there is no strong LD (*r* ^*2*^<0.8) between the genetically associated variants on chromosome 32, the presence of multiple highly associated SNPs across the 1.3 Mb region and their association with gene expression levels suggests that the entire region including several genes is important for SRMA susceptibility. While many typed variants show correlation with gene expression, there is one tag SNP (chr32:24,827,518) whose risk allele A is most strongly associated with increased expression of *DAPP1*, *LAMTOR3*, *DDIT4L* and *PPP3CA*. The distant *cis*-effect on expression of genes placed far away from each other may indicate a complex topology of the chromosomal locus with a possible locus control region(s) and common enhancers driving regulation of genes located on the opposite DNA strands.

The previously reported signal on chromosome 8 [27] was corroborated in our study, but we found no correlation between the SRMA SNPs and expression levels of the only nearby gene *VRK1*. Thereby, the identity of the gene affected in this locus in SRMA remains to be identified or the SNP could have its effect in a different tissue than PBMCs. Also, the progress in mapping non-coding genes including microRNA and lincRNA genes on the dog genome [57] may help to resolve this question in the future. The SRMA associated haplotype on chromosome 3 is correlated with increased expression of the *AP3B2* gene.

**Fig 8:**
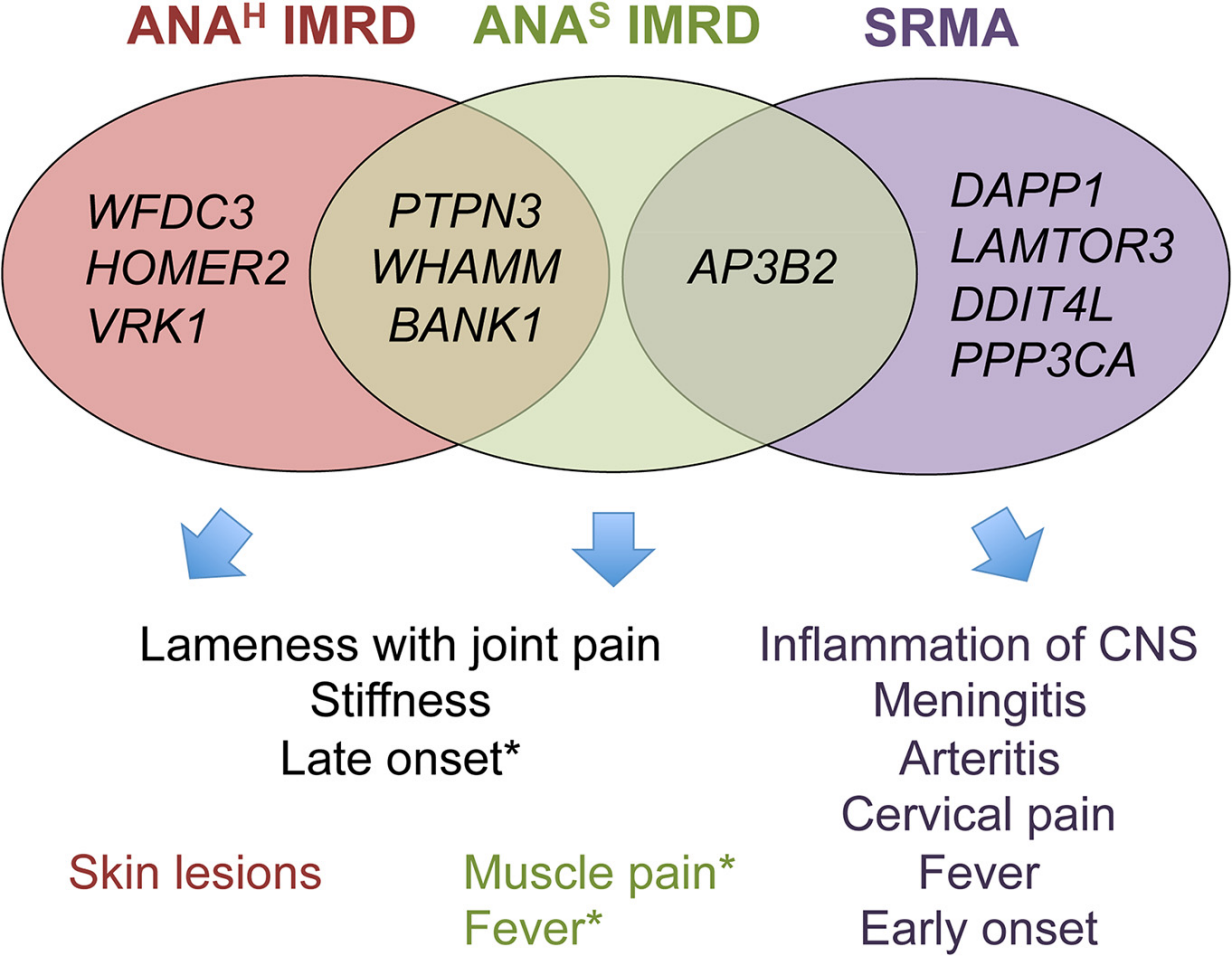
Venn diagram showing overlaps between clinicopathologic features and associated genes. * Muscle pain and fever occur in both ANA-positive groups, but were slightly more frequent in ANA^S^ dogs in the study cohort. ANA^S^ showed an earlier onset of disease than ANA^H^ (2 versus 3 years), while SRMA affected dogs at even a younger age (4–19 months).

Interestingly, there is little overlap between the genes associated with SRMA and IMRD, suggesting the involvement of largely distinct pathways in these phenotypes (**Fig 8**). While most of the 11 genes are widely expressed, there is a certain emphasis on the predominant brain, muscle and immune cells expression of SRMA genes *AP3B2*, *PPP3CA*, *DDIT4L*, *DAPP1* and *LAMTOR3* (**S7 Fig**), consistent with the involvement of the central nervous system and muscles seen in this disease. Moreover, two genes, *DDIT4L* and *PPP3CA*, show relatively strong association signals in human neuromuscular inflammatory conditions (**S10 Table**). Overall, for nine dog genes out of eleven, there is either evidence or at least a trend of association with various human autoimmune disorders, as can be viewed using the GRASP Search tool (http://apps.nhlbi.nih.gov/Grasp/Search.aspx)[58].

Without wishing to be bound by a theory, we hypothesize that primary inflammation triggered by as yet unknown environmental factors in SRMA-susceptible dogs is then maintained by the over-activated B cells producing high titers of IgA, often detected in the serum and cerebrospinal fluid (CSF) of SRMA patients [14, 56]. The enhanced function of *DAPP1*, *PPP3CA* and *LAMTOR3* acting downstream of the B cell receptor via MAPK signaling pathway may be responsible for the altered B cell reactions. Moreover, the arteritis of the blood vessels may lead to ischemia in CNS or myocardial infarction through decreased nutrient and oxygen supply by damaged arteries and may further induce an already genetically modulated *DDIT4L* in neurons or cardiac myocytes, which in turn inhibit mTOR signaling and activate autophagy (genes *LAMTOR3* and *AP3B2* code for adaptor proteins in the lysosome-endosome system) and lead to apoptosis or necrosis [59]. It is tempting to speculate here that the enhanced levels of brain-specific expression of the SRMA-associated genes could be responsible also for hyperesthesia, an extreme pain sensitivity condition mainly exhibited by cervical, neck and spinal pain, and always seen in SRMA but not IMRD dogs [14].

Of note, out of four genes associated with ANA^S^ IMRD only *AP3B2*, the gene also shared with SRMA, shows the highest expression levels in the brain. In contrast, the ANA^H^ genes showed a more diverse pattern of expression with the highest levels in the following tissues: blood (*BANK1* and *VRK1*), skin and kidney (*WFDC3*), muscle and kidney (*HOMER2*), heart, kidney and liver (*PTPN3*), muscle/heart, liver and kidney (*WHAMM*).

Many cellular pathways are interconnected and therefore it is difficult to predict the exact biological effect, especially when several genes are altered, so the signaling output of each affected gene in all three phenotypes, ANA^H^, ANA^S^ and SRMA, will depend on a particular tissue and cell context, the precise timing of expression and responsiveness to environmental inducers. Thus, for instance, the highest shift in expression levels associated with SRMA was a 2.5-fold up-regulation observed for the *DDIT4L* gene (**Fig 4H**), DNA-damage-inducible transcript 4-like, known to be induced by a variety of stress factors, including hypoxia [60]. Although the precise role of the encoded protein remains largely unknown, the function of DDIT4L in autoimmunity may be related to the negative regulation of mTOR [61]. While the inhibition of mTOR promotes generation of CD8^+^ memory T cells [62], at the same time it induces cell death by necrosis in the culture of U-937 monocytes [63], which may trigger an auto-inflammatory response.

In addition to the complexity of phenotypes relating to the different genes and genetic variants, the genomic architecture of almost all loci show a considerable complexity with SNPs showing long-range effects on gene regulation either alone or in conjunction with other SNPs. For example, the chromosome 3 top SNP (3:57,432,981) is a part of two different haplotypes present in different sub-phenotypes. On chromosome 32, a haplotype associated with ANA^S^ exerts a distant effect on a gene located in the other part of the locus, while SNPs located internally in the region affect four other genes in SRMA. It remains to be shown whether this type of complex regulatory structure will be a common finding in both canine and human disease, or an unusual event found here due to the potentially strong natural selection applied when only a handful of NSDTR dogs survived a canine distemper outbreak in the early 1900s [64] remains to be seen.

In conclusion, we identified functional changes in eleven genes associated with different sub-phenotypes of canine IMRD resembling human SLE, and with SRMA which share clinical signs with a group of human vasulitides including Kawasaki disease, Henoch-Schönlein purpura and Behçet’s disease. Based on the gene functions and pattern of expression, we hypothesize how different genetic factors, sometimes located in the same genomic region, may lead to diverse clinical manifestations. The common genes and pathways may account for overlapping manifestations, whereas alterations in the functions of certain tissue-specific or even ubiquitous genes may be linked to specific clinical signs. The strikingly complex pattern of genomic regulation suggests that one should keep an open mind to multiple variants causing disease when studying GWAS regions both in canine and human diseases. The utility of dog breeds in identification of disease genes underlying human complex diseases is demonstrated by the identification of a well-known human SLE gene, *BANK1*, and several novel genes. The novel genes warrant further study both in canine and human autoimmune disease.

## Materials and Methods

### Ethics statement

This study was performed in strict accordance with the guidelines of the EU directive 2010/63 on the protection of animals used for scientific purposes. The protocols were approved by the regional Ethical board for experimental animals in Uppsala, Sweden (Dnr C103/10 and C417/12), and Animal Ethical Committee of County Administrative Board of Southern Finland (ESAVI/6054/04.10.03/2012).

### Study population and diagnostic procedures

250 Nova Scotia duck tolling retrievers (NSDTRs) were included in this study, 52 of them classified as ANA-positive IMRD cases, 66 as SRMA cases and 132 as healthy controls. All dogs included were privately owned and samples were collected during 2002–2013. The dog pedigrees were verified to exclude closely related animals (littermates) from the analysis. Individual dog owners had consulted different veterinary clinics in Sweden and Finland. The inclusion criteria for IMRD ANA-positive dogs were musculoskeletal signs indicating a systemic rheumatic disorder, including stiffness mainly after rest, and pain from several joints of extremities. These signs had to be apparent for at least 14 days and were the main reason for the dog owner to visit the veterinary clinic. The examining veterinary physician suspected no other diseases in their diagnosis. All IMRD dogs should also display a positive IIF ANA test. Healthy controls were above seven years of age with no history of autoimmune disease. All study dogs were verified for relatedness and those related were excluded from the analysis.

ANA tests were analyzed with indirect immunofluorescence at the University Animal Hospital, Swedish University of Agricultural Sciences (SLU), Uppsala, Sweden using monolayers of HEp-2 cells fixed on glass slides (Immuno Concepts). The glass slides were examined by fluorescence microscopy and considered positive at a titer of ≥1:100. The visible nuclear fluorescence patterns could be divided into two groups; homogeneous (ANA^H^) or speckled (ANA^S^) patterns as previously described[8].

### DNA purification, PCR amplification of DLA regions and sequence analysis

Genomic DNA was purified from 200 μl of blood using Qiagen QIAamp DNA Blood Mini Kit (Qiagen) according to the manufacturer’s protocol.

DLA-DRB1,-DQA1 and DQB1 exon 2 were amplified by PCR as previously described[25].

DNA sequencing was performed using capillary electrophoresis on an Applied Biosystems 3730xl. BigDye Terminator v3.1 (Applied Biosystems) Sequencing of the purified PCR products was made in one direction, reverse for DLA-DRB1 and-DQA1 and forward for DLA-DQB1. Analysis of the nucleotide sequence was performed using MatchTools and MatchTools Navigator (Applied Biosystems) [25].

### Statistical analysis

Statistical analyses were performed using VassarStats (http://vassarstats.net/odds2x2.html). Odds ratios and p-values for each allele, haplotype and genotype were calculated using a 2x2 contingency table. The total number of cases and controls carrying a specific allele or genotype was compared with the cases and controls not carrying it. The same comparison was made for alleles as well as genotypes for the ANA-positive cases with homogeneous or speckled pattern and the controls. The total numbers of homozygous dogs was also compared in cases and controls.

### Next generation re-sequencing

To identify candidate variants, the five regions previously found associated with IMRD [27], spanning approximately 5Mb, were re-sequenced in nine NSDTR individuals (four ANA cases, two SRMA cases and three controls) using 385K custom designed capture arrays from Roche NimbleGen and 400–600 X coverage Illumina sequencing. The sequencing data was aligned with BWA (http://bio-bwa.sourceforge.net/) [65] and analyzed using SAMtools (http://samtools.sourceforge.net/) [66], BEDTools (http://code.google.com/p/bedtools/) [67], SEQscoring [33] (http://www.seqscoring.org/) and other in-house tools to discover variants (SNPs, indels and structural changes) in the genomic sequence between IMRD, SRMA and healthy control dogs. A total of 13,084 potential SNPs were detected and of these, 426 SNPs were located within or close (±5 bp) to a conserved element [39, 68]. 2780 possible InDels were detected, among those, 88 occurred within or close to a conserved element (±5 bp). To identify structural variations, such as larger insertions, CNV or deletions, SEQscoring was used to calculate coverage differences between cases and controls. We did not identify any structural variants that differed between cases and controls. Sequencing data was deposited in European Nucleotide Archive (ENA) (Study accession: PRJEB6494, available at: http://www.ebi.ac.uk/ena/data/view/PRJEB6494).

### SNP selection for genotyping and association analysis

308 SNPs for five loci (chromosome 3, 8, 11, 24 and 32) were chosen from the re-sequencing data. SNPs were chosen based on the following criteria: difference in allele frequency in cases compared to controls, positioned in either protein coding regions, 5’ UTR or 3’ UTR and located within non-coding conserved elements. Conserved elements were identified using comparative sequence analysis based on the analysis of 29 mammals using SiPhy [39, 68]. These SNPs were genotyped by GoldenGate Genotyping Assay. PLINK [69] (http://pngu.mgh.harvard.edu/purcell/plink/) was used to analyze the markers with a MAF >0.05 and a call rate >0.75. Total genotyping rate was 97%.

### RNA extraction and cDNA synthesis

Peripheral blood was drawn from 165 healthy NSDTR dogs directly in Tempus Blood RNA tubes (Applied Biosystems) and kept on ice during transportation. For isolation of total RNA from different dog tissues, fresh tissue samples from euthanized dogs were immediately immersed in the TRIZOL solution (Invitrogen) and RNA and DNA were purified according to the manufacture’s protocol. Total RNA from blood was purified using the Tempus Spin RNA Isolation Reagent kit (Applied Biosystems) according to the manufacturer’s instructions, and the quantity and the quality of RNA was assessed by NanoDrop ND-1000 spectrophotometer (Thermo Scientific). In parallel, genomic DNA was purified for each sample and genotyped using pyrosequencing or direct Sanger sequencing with the primers shown in **S11 Table**. cDNA synthesis was performed in 20 μL at 42°C for 80 min using 2 μg of total RNA, 5 μM oligo-dT primer, MuLV transcriptase, RNase inhibitor in the buffer supplemented with 5 mM MgCl_2_ and 1 mM dNTPs. All reagents were purchased from Applied Biosystems. The reaction was terminated by heating for 5 min at 95°C and diluted to 25 ng/μl.

### Quantitative RT-PCR

Gene expression was measured by quantitative real-time PCR on 7900HT Sequence Detector (Applied Biosystems) with SDS 2.3 software using SYBR Green for signal detection. Gene-specific primers and annealing temperatures are shown in **S12 Table** in accordance with the guidelines for the minimum information for publication of quantitative real-time PCR experiments (MIQE) [70]. The regions for primers’ design were selected to target all known transcripts for a particular gene and thus allow to measure the total gene expression. In order to avoid amplification from genomic DNA, primers were located to cover either several exons separated by long introns or exon/exon junctions and further verified by BLAST search. The PCR conditions were optimized for each primer set prior to qPCR, and the specificity of amplification was verified by the post-PCR analysis of melting curves and agarose gel analysis. Initial denaturation at 95°C for 5 min was followed by 45 cycles (95°C for 15s, annealing at primer-specific Tm for 15s and 72°C for 25s). PCR buffer (Invitrogen) was supplemented with 1.5 mM MgCl_2_, 200 μM of each dNTPs, 0.2 μM of each primer, SYBRGreen (Molecular Probes), 15 ng of cDNA and 0.5 U of Platinum Taq polymerase (Invitrogen). The reaction was carried out in 20 μL on a MicroAmp Optical 384-well reaction plate (Applied Biosytems). Expression levels were normalized to the reference gene *TBP* using the comparative 2^-ΔCt^-method [71]. All experiments were run in triplicate. Correlation of gene expression with genotypes and haplotypes was performed using one-way ANOVA tests in PRISM 6 (GraphPad Software).

### Luciferase reporter assay

The dog 550 bp genomic fragment containing SNP chr8:68,708,503 was amplified by PCR and cloned in front of the minimal promoter in the pGL4.26 reporter vector (Promega). After sequence validation, the plasmids were purified with EndoFree Plasmid Maxi Kit (Qiagen). The transfection of K562 cells was performed in the 24-well plates as follows: 7x10^5^ cells/well were seeded 24 hours before transfection in the RPMI-1640 medium supplemented with L-glutamine and 10% of heat-inactivated bovine serum. 750 ng of the reporter plasmid and 50 ng of the pRL-TK (Promega) normalization vector were transfected into each well by Lipofectamine 2000 (Invitrogen) according to the manufacture’s protocol. Twenty-four hours after transfection, cells were additionally stimulated with 20 ng/ml of PMA for 10 hours, then harvested and assayed for the Firefly and Renilla luciferase activities with the Dual-Luciferase Reporter Assay System (Promega). The experiment was repeated three times with four technical replicates for each plasmid.

## Supporting Information

S1 Fig The comparative expression levels of genes from chromosome 32 locus. The gene expression was measured in the total RNA purified from canine blood and normalized to the levels of the *TBP* gene.
(PDF)

S2 Fig Tissue-specific expression of *FSD2* in dogs analyzed by RT-PCR in total RNA purified from different tissues. The gene is highly expressed in heart, skeletal muscles, testis, skin, kidney and cartilage, and in the canine MDCK cell line.
(PDF)

S3 Fig The *HOMER2* gene expression in dog blood cells. The best of the genotyped SNPs associated with *HOMER2* expression is 3:57564331 (not associated with SLE) (A). Expression of *HOMER2* stratified by the 2-SNP haplotype 57432981–57546568. The associated with SLE haplotype includes the non-synonymous substitution (Thr->Ala) and display a trend, not statistically significant though due to sample size, towards gene down-regulation, and is shown in red color, the protective haplotype—in blue color (B).
(PDF)

S4 Fig Expression of *WHAMM* stratified by the 2-SNP haplotype 57432981–57484486. (PDF)

S5 Fig Common structure of the HOMER family proteins (A). Two major domains EVH1 and dimerization coiled coil region are shown. Partial alignment of the EVH1 domain including β4 to β6 sheets shown for all three proteins: HOMER1 (**B**), HOMER2 (**C**), and HOMER3 (**D**). The non-synonymous SNP in *HOMER2* changing conserved Thr to Ala and the corresponding amino acid in the EVH1 domain of HOMER1 and HOMER3 is enclosed in red squares. The protein alignment was performed by Vertebrate Multiz Alignment & Conservation (44 Species) at http://www.genome.ucsc.edu/. Hydrophobic core residue is marked by an asterisk, the two amino acids critical for the peptide binding site are marked with diamonds. The amino acid substitution in HOMER2 protein does not affect Thr-phosphorylation as analyzed by NetPhos 2.0 at http://www.cbs.dtu.dk/services/NetPhos/ and PhosphoMotif Finder at http://www.hprd.org/PhosphoMotif_finder.
(PDF)

S6 Fig D’-plot for the associated region on chromosome 8. The top three associated SNPs, two for ANA^H^ with MHC haplotype, 8:68708503 and 8:68712185, and one SNP for SRMA 8:68726546 are labeled with green ovals.
(PDF)

S7 Fig Gene expression measured in canine tissues by RNA-seq [57]. The data were uploaded from http://genome.ucsc.edu/cgi-bin/hgHubConnect‘‘Broad Improved Canine Annotation v1”). *AP3B2* is not detected in heart, *DDIT4L* in skin and *BANK1* in liver, all other genes have broad expression. The small bars indicating low expression levels could be invisible due to the graph scale.
(PDF)

S1 Table Diagnostic information and DLA-DRB1,-DQA1 and-DQB1 alleles, haplotypes and genotypes for all dogs included in the study. (PDF)

S2 Table Allele frequencies and statistical results in the NSDTR population including all ANA-positive cases, ANA^S^, ANA^H^ and controls. Five different DRB1, four DQA1 and five DQB1 alleles where identified.
(PDF)

S3 Table Haplotype frequencies in the NSDTR population reveal an associated haplotype for ANA^S^ dogs (DLA-DRB1*00601/DQA1*005011/DQB1*02001). (PDF)

S4 Table Homozygosity at MHC class II observed in the NSDTR population including all genotypes with and without the risk genotype 2. (PDF)

S5 Table Positions of all SNPs genotyped and p-values for genetic association, minor allele (A1) and major allele (A2) for each phenotype studies. (PDF)

S6 Table Association of differential expression of *WFDC3* with genotyped variants on chromosome 24. (PDF)

S7 Table Association of differential expression of genes with genotyped variants across the chromosome 32 locus. (PDF)

S8 Table Association of differential expression of genes with genotyped variants on chromosome 3. (PDF)

S9 Table Clinical manifestations in ANA positive NSDTRs. (PDF)

S10 Table The association signals for the dog genes obtained in various human genome-wide association studies and retrieved by the GRASP search tool. The cut-off p-value is 0.001. The results only for autoimmune diseases (red) and neuroinflammatory conditions (blue) are presented.
(XLSX)

S11 Table Primer pairs used for genotyping in expression samples. (PDF)

S12 Table Primers for qRT-PCR. (PDF)
