# Supplementary figures and images for: Multiple Changes of Gene Expression and Function Reveal Genomic and Phenotypic Complexity in SLE-like Disease
Source: PLoS Genet. 2015 Jun 9;11(6):e1005248. doi: 10.1371/journal.pgen.1005248 (PMC4461293; doi:10.1371/journal.pgen.1005248)

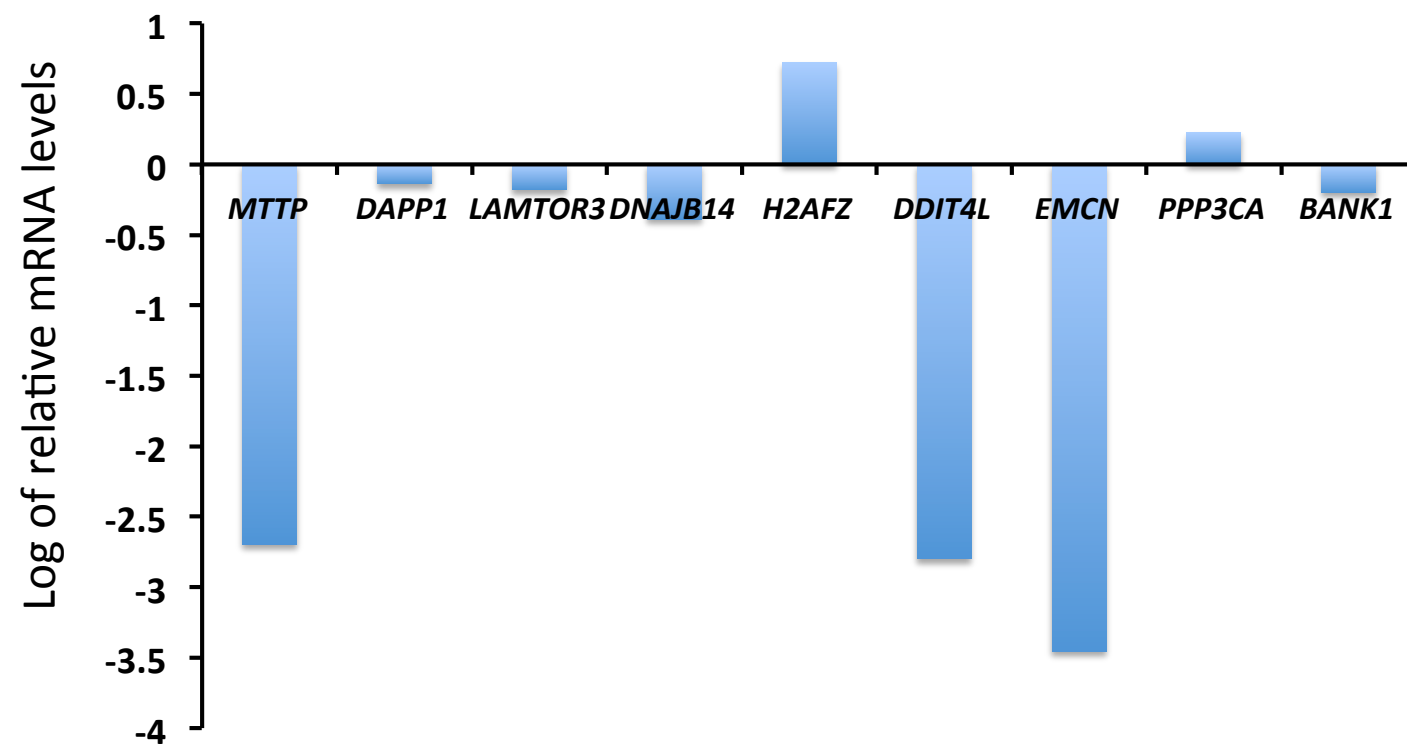

Supplement: S1 Fig — The gene expression was measured in the total RNA purified from canine blood and normalized to the levels of the TBP gene. (PDF) [file pgen.1005248.s001.pdf]

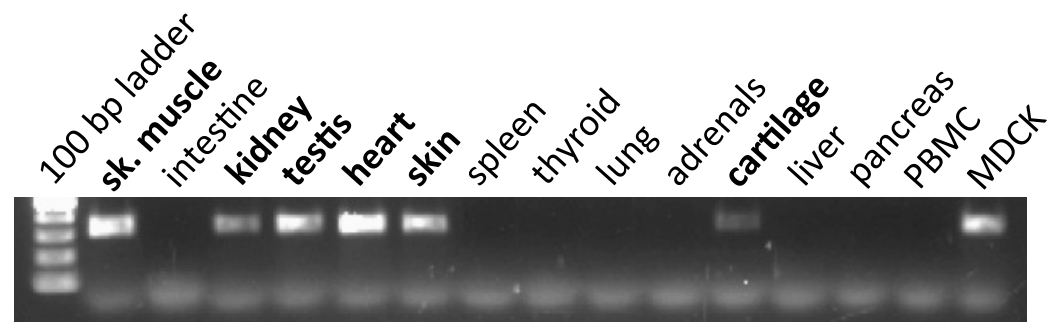

Supplement: S2 Fig — The gene is highly expressed in heart, skeletal muscles, testis, skin, kidney and cartilage, and in the canine MDCK cell line. (PDF) [file pgen.1005248.s002.pdf]

**A***HOMER2*,  $P_{ANOVA}=0.0924$ 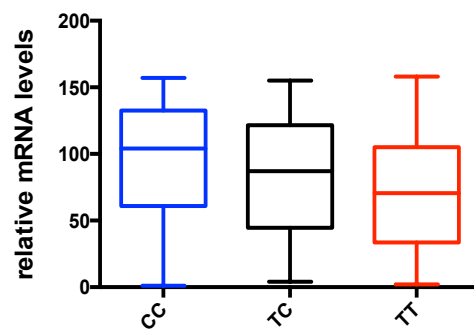

3:57564331

**B***HOMER2*,  $P_{ANOVA}=0.1905$ 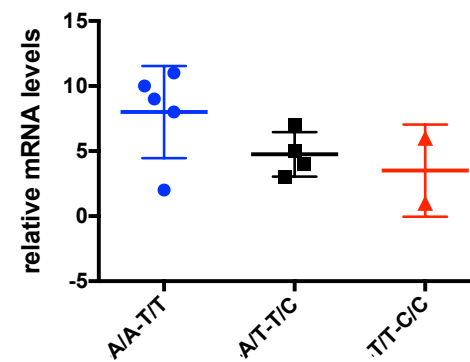

2 SNP haplotype:  
57432981-57546568

Supplement: S3 Fig — The best of the genotyped SNPs associated with HOMER2 expression is 3:57564331 (not associated with SLE) (A). Expression of HOMER2 stratified by the 2-SNP haplotype 57432981–57546568. The associated with SLE haplotype includes the non-synonymous substitution (Thr->Ala) and display a trend, not statistically significant though due to sample size, towards gene down-regulation, and is shown in red color, the protective haplotype—in blue color (B). (PDF) [file pgen.1005248.s003.pdf]

*WHAMM*,  $P_{ANOVA}=0.016$

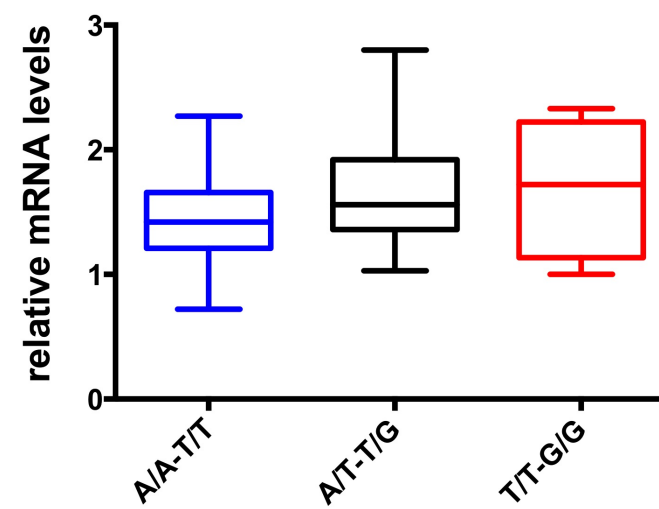

2 SNP haplotype:  
57432981-57484486

Supplement: S4 Fig — (PDF) [file pgen.1005248.s004.pdf]

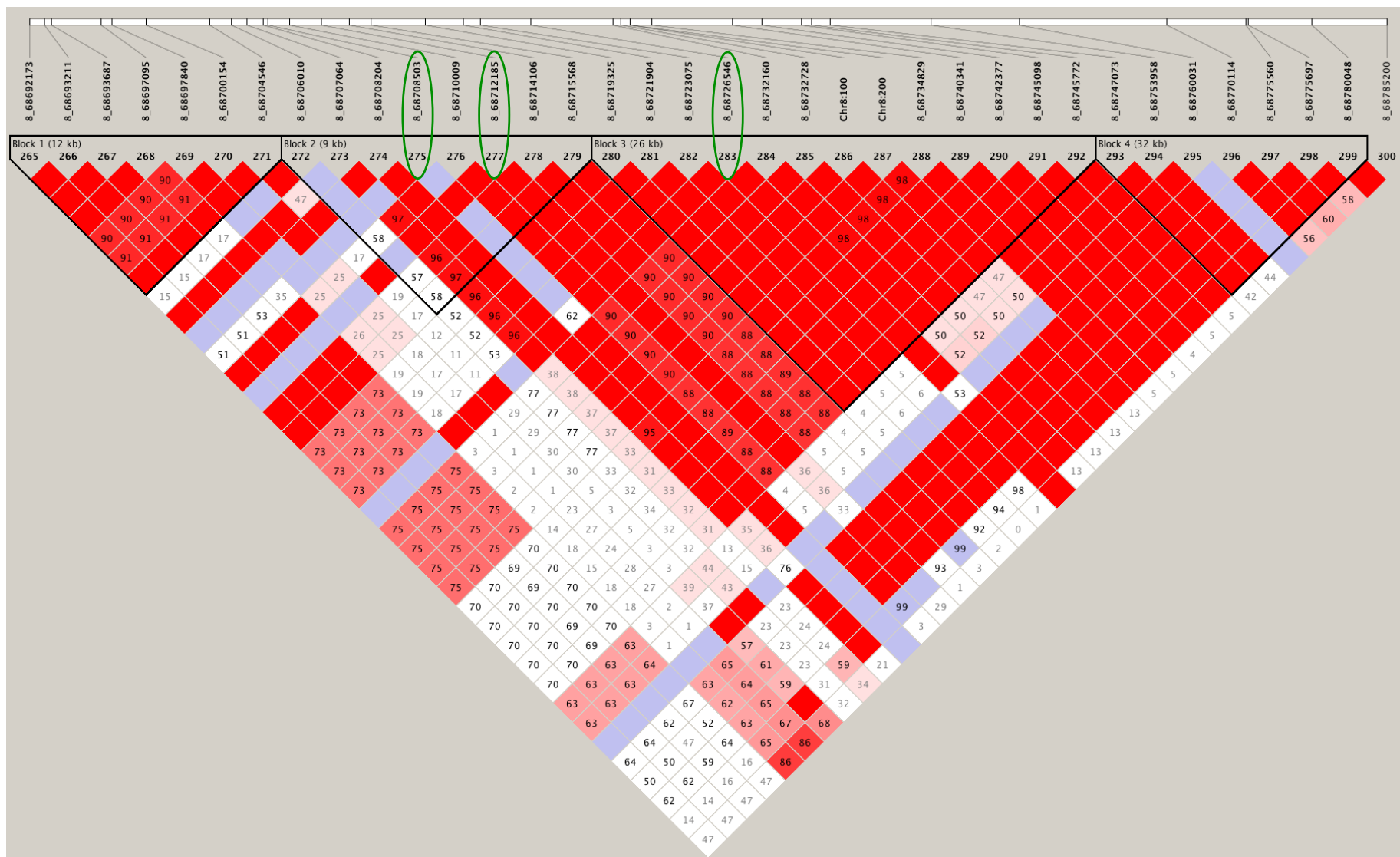

Supplement: S6 Fig — The top three associated SNPs, two for ANAH with MHC haplotype, 8:68708503 and 8:68712185, and one SNP for SRMA 8:68726546 are labeled with green ovals. (PDF) [file pgen.1005248.s006.pdf]

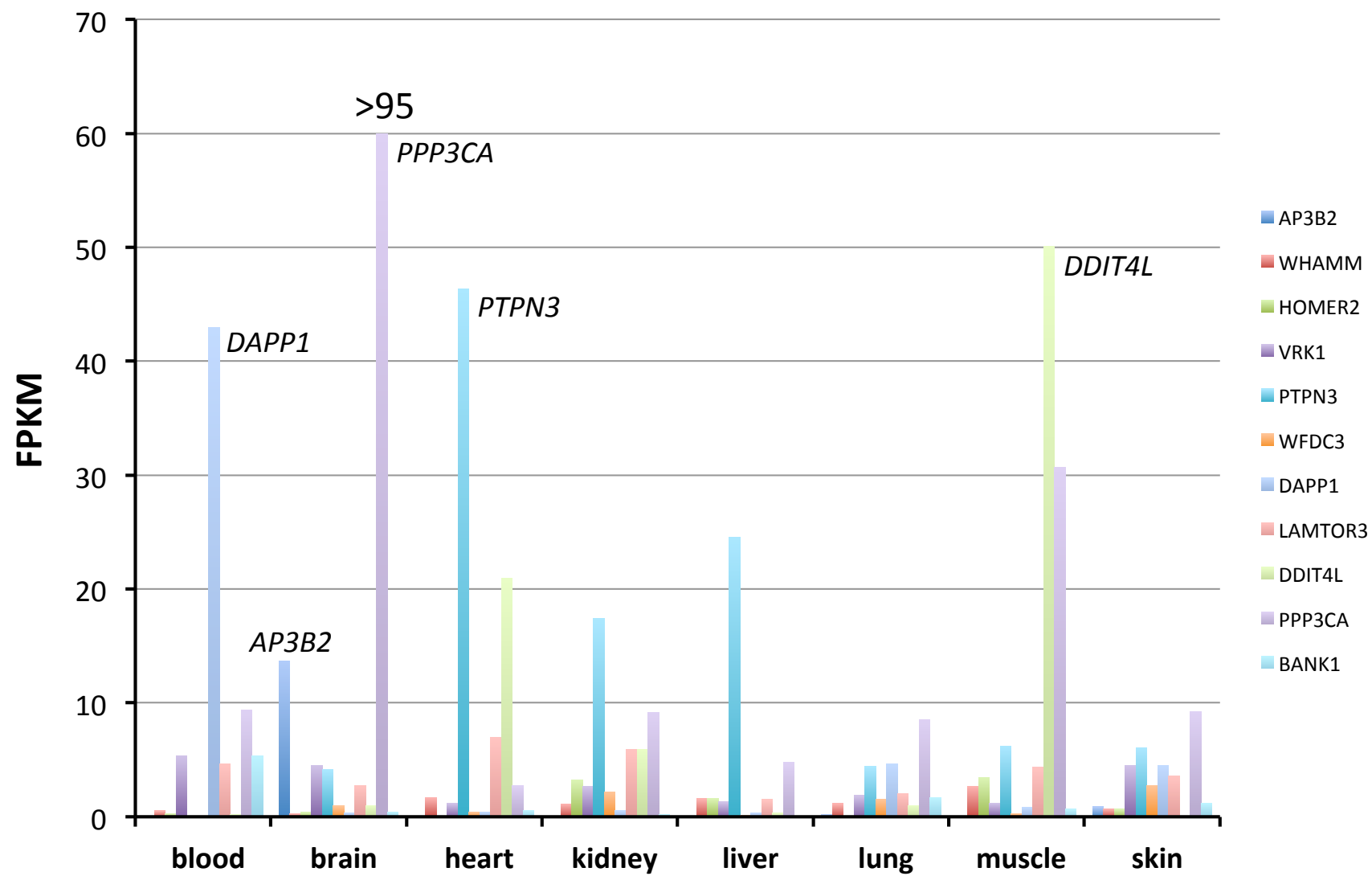

Supplement: S7 Fig — The data were uploaded from http://genome.ucsc.edu/cgi-bin/hgHubConnect‘‘Broad Improved Canine Annotation v1”). AP3B2 is not detected in heart, DDIT4L in skin and BANK1 in liver, all other genes have broad expression. The small bars indicating low expression levels could be invisible due to the graph scale. (PDF) [file pgen.1005248.s007.pdf]
