# Supplementary material for: Multiple Changes of Gene Expression and Function Reveal Genomic and Phenotypic Complexity in SLE-like Disease
Source: PLoS Genet. 2015 Jun 9;11(6):e1005248. doi: 10.1371/journal.pgen.1005248 (PMC4461293; doi:10.1371/journal.pgen.1005248)
Supplement: S5 Fig — Two major domains EVH1 and dimerization coiled coil region are shown. Partial alignment of the EVH1 domain including β4 to β6 sheets shown for all three proteins: HOMER1 (B), HOMER2 (C), and HOMER3 (D). The non-synonymous SNP in HOMER2 changing conserved Thr to Ala and the corresponding amino acid in the EVH1 domain of HOMER1 and HOMER3 is enclosed in red squares. The protein alignment was performed by Vertebrate Multiz Alignment & Conservation (44 Species) at http://www.genome.ucsc.edu/. Hydrophobic core residue is marked by an asterisk, the two amino acids critical for the peptide binding site are marked with diamonds. The amino acid substitution in HOMER2 protein does not affect Thr-phosphorylation as analyzed by NetPhos 2.0 at http://www.cbs.dtu.dk/services/NetPhos/ and PhosphoMotif Finder at http://www.hprd.org/PhosphoMotif_finder. (PDF) [file pgen.1005248.s005.pdf]

HOMER family protein structure

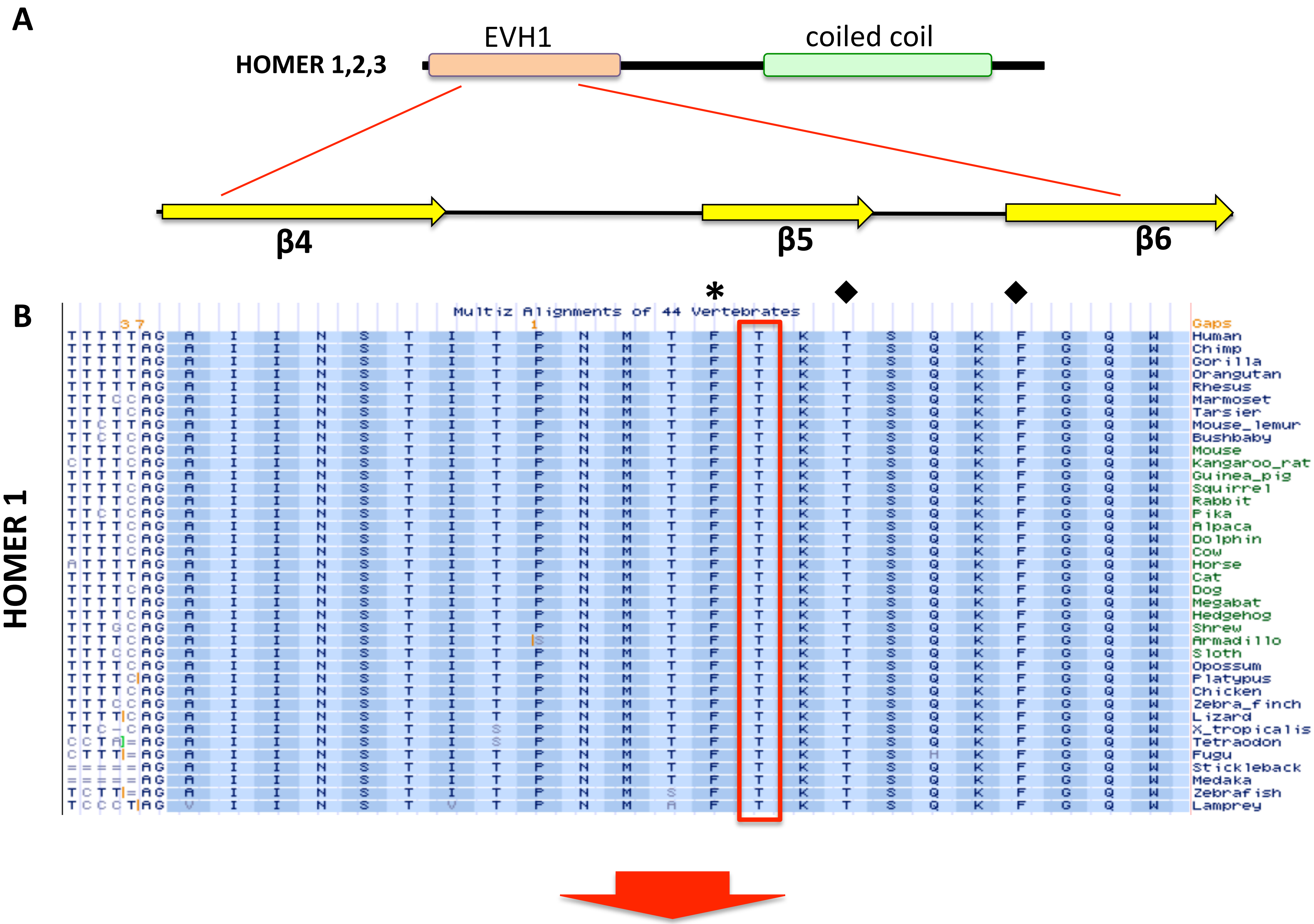

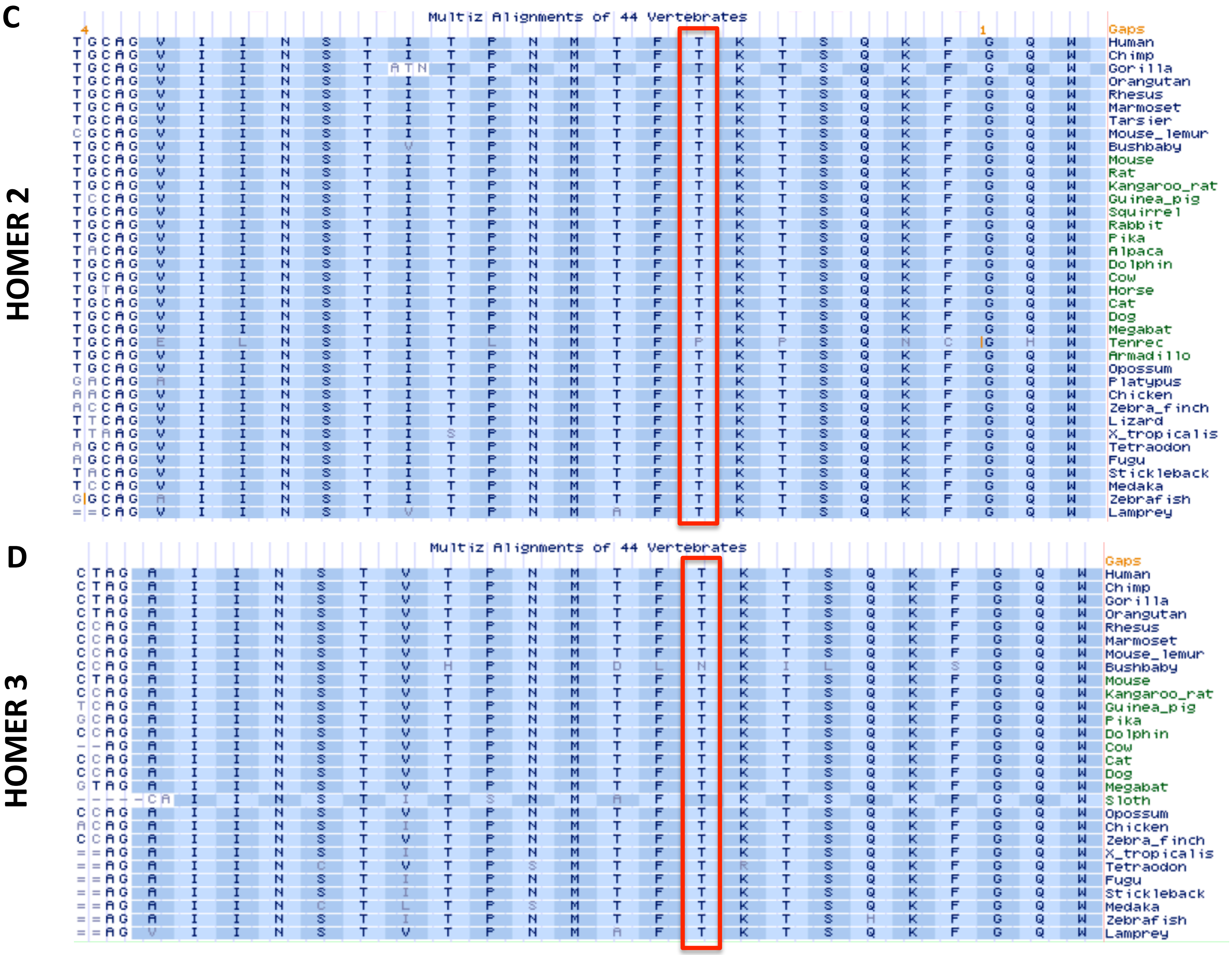
C
HOMER 2
Multiz Alignments of 44 Vertebrates
Gaps
Human
Chimp
Gorilla
Orangutan
Rhesus
Marmoset
Tarsier
Mouse_lemur
Bushbaby
Mouse
Rat
Kangaroo_rat
Guinea_pig
Squirrel
Rabbit
Pika
Alpaca
Dolphin
Cow
Horse
Cat
Dog
Megabat
Tenrec
Armadillo
Opossum
Platypus
Chicken
Zebra_finch
Lizard
X_tropicalis
Tetraodon
Fugu
Stickleback
Medaka
Zebrafish
Lamprey
D
HOMER 3
Multiz Alignments of 44 Vertebrates
Gaps
Human
Chimp
Gorilla
Orangutan
Rhesus
Marmoset
Mouse_lemur
Bushbaby
Mouse
Kangaroo_rat
Guinea_pig
Pika
Dolphin
Cow
Cat
Dog
Megabat
Sloth
Opossum
Chicken
Zebra_finch
X_tropicalis
Tetraodon
Fugu
Stickleback
Medaka
Zebrafish
Lamprey
