# Supplementary material for: Multiple Changes of Gene Expression and Function Reveal Genomic and Phenotypic Complexity in SLE-like Disease
Source: PLoS Genet. 2015 Jun 9;11(6):e1005248. doi: 10.1371/journal.pgen.1005248 (PMC4461293; doi:10.1371/journal.pgen.1005248)
Supplement: S1 Table — (PDF) [file pgen.1005248.s008.pdf]

Table S1. Diagnostic information and DLA-DRB1, -DQA1 and -DQB1 alleles, haplotypes and genotypes for all dogs included in the study.

ID	Status	ANA	DRB1	DQA1	DQB1	DRB1	DQA1	DQB1	Haplotype	Genotype
1	Case	ANA	01502	00601	02301	01502	00601	02301	**1.1**	1
2	Case	ANA	01502	00601	02301	01502	00601	02301	**1.1**	1
3	Case	ANA	00601	005011	02001	01502	00601	02301	**1.2**	2
4	Case	ANA	01501	00601	00301	01502	00601	02301	**1.3**	3
5	Case	ANA	02301	00301	00501	01502	00601	02301	**1.5**	5
6	Case	ANA	00601	005011	02001	00601	005011	02001	**2.2**	6
7	Case	ANA^H	01502	00601	02301	01502	00601	02301	**1.1**	1
8	Case	ANA^H	01502	00601	02301	01502	00601	02301	**1.1**	1
9	Case	ANA^H	01502	00601	02301	01502	00601	02301	**1.1**	1
10	Case	ANA^H	01502	00601	02301	01502	00601	02301	**1.1**	1
11	Case	ANA^H	01502	00601	02301	01502	00601	02301	**1.1**	1
12	Case	ANA^H	01502	00601	02301	01502	00601	02301	**1.1**	1
13	Case	ANA^H	01502	00601	02301	01502	00601	02301	**1.1**	1
14	Case	ANA^H	01502	00601	02301	01502	00601	02301	**1.1**	1
15	Case	ANA^H	01502	00601	02301	01502	00601	02301	**1.1**	1
16	Case	ANA^H	00601	005011	02001	01502	00601	02301	**1.2**	2
17	Case	ANA^H	01501	00601	00301	01502	00601	02301	**1.3**	3
18	Case	ANA^H	01501	00601	00301	01502	00601	02301	**1.3**	3
19	Case	ANA^H	01501	00601	00301	01502	00601	02301	**1.3**	3
20	Case	ANA^H	01501	00601	00301	01502	00601	02301	**1.3**	3
21	Case	ANA^H	01501	00601	00301	01502	00601	02301	**1.3**	3
22	Case	ANA^H	01501	00601	00301	01502	00601	02301	**1.3**	3
23	Case	ANA^H	01502	00601	02301	02301	00301	00501	**1.5**	5
24	Case	ANA^H	00601	005011	02001	00601	005011	02001	**2.2**	6
25	Case	ANA^H	00601	005011	02001	00601	005011	02001	**2.2**	6
26	Case	ANA^H	00601	005011	02001	01501	00601	00301	**2.3**	7
27	Case	ANA^H	01501	00601	00301	01501	00601	00301	**3.3**	9
28	Case	ANA^H	01501	00601	00301	01501	00601	00301	**3.3**	9
29	Case	ANA^H	01501	00601	00301	01501	00601	00301	**3.3**	9
30	Case	ANA^H	01501	00601	00301	01501	00601	00301	**3.3**	9
31	Case	ANA^H	01501	00601	00301	01501	00601	00301	**3.3**	9
32	Case	ANA^H	01502	00601	02301	01502	00601	02301	**1.1**	1
33	Case	ANA^S	00601	005011	02001	01502	00601	02301	**1.2**	2
34	Case	ANA^S	00601	005011	02001	01502	00601	02301	**1.2**	2
35	Case	ANA^S	00601	005011	02001	01502	00601	02301	**1.2**	2
36	Case	ANA^S	02301	00301	00501	01502	00601	02301	**1.5**	5
37	Case	ANA^S	00601	005011	02001	00601	005011	02001	**2.2**	6
38	Case	ANA^S	00601	005011	02001	00601	005011	02001	**2.2**	6
39	Case	ANA^S	00601	005011	02001	00601	005011	02001	**2.2**	6
40	Case	ANA^S	00601	005011	02001	00601	005011	02001	**2.2**	6
41	Case	ANA^S	00601	005011	02001	00601	005011	02001	**2.2**	6
42	Case	ANA^S	00601	005011	02001	00601	005011	02001	**2.2**	6
43	Case	ANA^S	00601	005011	02001	00601	005011	02001	**2.2**	6
44	Case	ANA^S	00601	005011	02001	00601	005011	02001	**2.2**	6
45	Case	ANA^S	00601	005011	02001	00601	005011	02001	**2.2**	6
46	Case	ANA^S	00601	005011	02001	00601	005011	02001	**2.2**	6
47	Case	ANA^S	00601	005011	02001	00601	005011	02001	**2.2**	6

48	Case	ANA^{S}	00601	005011	02001	00601	005011	02001	**2.2**	6
49	Case	ANA^{S}	00601	005011	02001	00601	005011	02001	**2.2**	6
50	Case	ANA^{S}	00601	005011	02001	00601	005011	02001	**2.2**	6
51	Case	ANA^{S}	00601	005011	02001	00601	005011	02001	**2.2**	6
52	Case	ANA^{S}	00601	005011	02001	00601	005011	02001	**2.2**	6
53	Case	ANA^{S}	00601	005011	02001	00601	005011	02001	**2.2**	6
54	Case	ANA^{S}	00601	005011	02001	00601	005011	02001	**2.2**	6
55	Case	ANA^{S}	00601	005011	02001	00601	005011	02001	**2.2**	6
56	Case	ANA^{S}	02301	00301	00501	00601	005011	02001	**2.5**	8
57	Case	ANA^{S}	01501	00601	00301	01501	00601	00301	**3.3**	9
58	Case	ANA^{S}	00601	005011	02001	00601	005011	02001	**2.2**	6
59	Case	ANA^{S}	00601	005011	02001	00601	005011	02001	**2.2**	6
60	Control	Negative	01502	00601	02301	01502	00601	02301	**1.1**	1
61	Control	Negative	01502	00601	02301	01502	00601	02301	**1.1**	1
62	Control	Negative	01502	00601	02301	01502	00601	02301	**1.1**	1
63	Control	Negative	01502	00601	02301	01502	00601	02301	**1.1**	1
64	Control	Negative	01502	00601	02301	01502	00601	02301	**1.1**	1
65	Control	Negative	01502	00601	02301	01502	00601	02301	**1.1**	1
66	Control	Negative	01502	00601	02301	00601	005011	02001	**1.2**	2
67	Control	Negative	01502	00601	02301	00601	005011	02001	**1.2**	2
68	Control	Negative	01502	00601	02301	00601	005011	02001	**1.2**	2
69	Control	Negative	01502	00601	02301	00601	005011	02001	**1.2**	2
70	Control	Negative	01502	00601	02301	00601	005011	02001	**1.2**	2
71	Control	Negative	01502	00601	02301	00601	005011	02001	**1.2**	2
72	Control	Negative	01502	00601	02301	00601	005011	02001	**1.2**	2
73	Control	Negative	01502	00601	02301	00601	005011	02001	**1.2**	2
74	Control	Negative	01502	00601	02301	00601	005011	02001	**1.2**	2
75	Control	Negative	01502	00601	02301	00601	005011	02001	**1.2**	2
76	Control	Negative	01502	00601	02301	00601	005011	02001	**1.2**	2
77	Control	Negative	01502	00601	02301	00601	005011	02001	**1.2**	2
78	Control	Negative	01502	00601	02301	00601	005011	02001	**1.2**	2
79	Control	Negative	01502	00601	02301	00601	005011	02001	**1.2**	2
80	Control	Negative	01502	00601	02301	00601	005011	02001	**1.2**	2
81	Control	Negative	01502	00601	02301	00601	005011	02001	**1.2**	2
82	Control	Negative	01502	00601	02301	00601	005011	02001	**1.2**	2
83	Control	Negative	01502	00601	02301	00601	005011	02001	**1.2**	2
84	Control	Negative	01502	00601	02301	01501	00601	00301	**1.3**	3
85	Control	Negative	01502	00601	02301	01501	00601	00301	**1.3**	3
86	Control	Negative	01502	00601	02301	01501	00601	00301	**1.3**	3
87	Control	Negative	01502	00601	02301	01501	00601	00301	**1.3**	3
88	Control	Negative	01502	00601	02301	01501	00601	00301	**1.3**	3
89	Control	Negative	01502	00601	02301	01501	00601	00301	**1.3**	3
90	Control	Negative	01502	00601	02301	01501	00601	00301	**1.3**	3
91	Control	Negative	01502	00601	02301	01501	00601	00301	**1.3**	3
92	Control	Negative	01502	00601	02301	01501	00601	00301	**1.3**	3
93	Control	Negative	01502	00601	02301	01501	00601	00301	**1.3**	3
94	Control	Negative	01502	00601	02301	01501	00601	00301	**1.3**	3
95	Control	Negative	01502	00601	02301	00401	00201	01501	**1.4**	4
96	Control	Negative	01502	00601	02301	02301	00301	00501	**1.5**	5
97	Control	Negative	01502	00601	02301	02301	00301	00501	**1.5**	5
98	Control	Negative	01502	00601	02301	02301	00301	00501	**1.5**	5
99	Control	Negative	00601	005011	02001	00601	005011	02001	**2.2**	6

100	Control	Negative	00601	005011	02001	00601	005011	02001	**2.2**	6
101	Control	Negative	00601	005011	02001	00601	005011	02001	**2.2**	6
102	Control	Negative	00601	005011	02001	00601	005011	02001	**2.2**	6
103	Control	Negative	00601	005011	02001	00601	005011	02001	**2.2**	6
104	Control	Negative	00601	005011	02001	00601	005011	02001	**2.2**	6
105	Control	Negative	00601	005011	02001	00601	005011	02001	**2.2**	6
106	Control	Negative	00601	005011	02001	00601	005011	02001	**2.2**	6
107	Control	Negative	00601	005011	02001	00601	005011	02001	**2.2**	6
108	Control	Negative	00601	005011	02001	01501	00601	00301	**2.3**	7
109	Control	Negative	00601	005011	02001	01501	00601	00301	**2.3**	7
110	Control	Negative	00601	005011	02001	01501	00601	00301	**2.3**	7
111	Control	Negative	00601	005011	02001	01501	00601	00301	**2.3**	7
112	Control	Negative	00601	005011	02001	01501	00601	00301	**2.3**	7
113	Control	Negative	00601	005011	02001	01501	00601	00301	**2.3**	7
114	Control	Negative	00601	005011	02001	01501	00601	00301	**2.3**	7
115	Control	Negative	00601	005011	02001	01501	00601	00301	**2.3**	7
116	Control	Negative	00601	005011	02001	02301	00301	00501	**2.5**	8
117	Control	Negative	00601	005011	02001	02301	00301	00501	**2.5**	8
118	Control	Negative	00601	005011	02001	02301	00301	00501	**2.5**	8
119	Control	Negative	01501	00601	00301	01501	00601	00301	**3.3**	9
120	Control	Negative	01501	00601	00301	01501	00601	00301	**3.3**	9
121	Control	Negative	01501	00601	00301	02301	00301	00501	**3.5**	10
122	Control	Negative	01501	00601	00301	02301	00301	00501	**3.5**	10

* Different colors mark the haplotypes
