# Supplementary material for: Multiple Changes of Gene Expression and Function Reveal Genomic and Phenotypic Complexity in SLE-like Disease
Source: PLoS Genet. 2015 Jun 9;11(6):e1005248. doi: 10.1371/journal.pgen.1005248 (PMC4461293; doi:10.1371/journal.pgen.1005248)
Supplement: S2 Table — Five different DRB1, four DQA1 and five DQB1 alleles where identified. (PDF) [file pgen.1005248.s009.pdf]

Table S2. Allele frequencies and statistical results in the NSDTR population including all ANA-positive cases, ANA^S, ANA^H and controls. Five different DRB1, four DQA1 and five DQB1 alleles where identified.

Allele	ANA % (2n=118)	ANA^H % (2n=52)	ANA^S % (2n=54)	Controls % (2n=126)	Total population % (2n=244)	OR	P-value
DRB1*00401	0.0	0.0	0.0	0.8	0.4	-	-
DRB1*00601	46.6	11.5	**85.2**	**37.3**	41.8	9.7	<0.0001
DRB1*01501	16.9	**32.7**	3.7	**19.8**	18.4	2.0	0.10
DRB1*01502	33.1	**53.8**	7.4	**35.7**	34.4	2.1	0.039
DRB1*02301	3.4	1.9	3.7	6.3	4.9	-	-
DQA1*00601	50.0	**86.5**	11.1	**55.6**	52.9	5.1	0.00017
DQA1*05011	46.6	11.5	**85.2**	**37.3**	41.8	9.7	<0.0001
DQA1*00201	0.0	0.0	0.0	0.8	0.4	-	-
DQA1*00301	3.4	1.9	3.7	6.3	4.9	-	-
DQB1*02301	33.1	**53.8**	7.4	**35.7**	34.4	2.1	0.039
DQB1*02001	46.6	11.5	**85.2**	**37.3**	41.8	9.7	<0.0001
DQB1*00301	16.9	**32.7**	3.7	**19.8**	18.4	2.0	0.1
DQB1*01501	0.0	0.0	0.0	0.8	0.4	-	-
DQB1*00501	3.4	1.9	3.7	6.3	4.9	-	-

S= Speckled, H=Homogeneous

Bold indicate between what groups the largest difference in allele frequencies occurred and where statistics were performed (OR and P-values).
