# Supplementary material for: Multiple Changes of Gene Expression and Function Reveal Genomic and Phenotypic Complexity in SLE-like Disease
Source: PLoS Genet. 2015 Jun 9;11(6):e1005248. doi: 10.1371/journal.pgen.1005248 (PMC4461293; doi:10.1371/journal.pgen.1005248)
Supplement: S3 Table — (PDF) [file pgen.1005248.s010.pdf]

Table S3. Haplotype frequencies in the NSDTR population reveal an associated haplotype for ANA^S dogs (DLA-DRB1*00601/DQA1*005011/DQB1*02001).

S= Speckled, H=Homogeneous

No	Haplotype DRB1/DQA1/DQB1	ANA % (2n=118)	ANA^H % (2n=52)	ANA^S % (2n=54)	Controls % (2n=126)	Total population % (2n=244)	OR	P-value
1	01502/00601/02301	33.1	**53.8**	7.4	**35.7**	34.4	2.1	0.039
2	00601/005011/02001	46.6	11.5	**85.2**	**37.3**	41.8	9.7	<0.0001
3	01501/00601/00301	16.9	**32.7**	3.7	**19.8**	18.4	2.0	0.10
4	00401/00201/01501	0.0	0.0	0.0	0.8	0.4	-	-
5	02301/00301/00501	3.4	1.9	3.7	6.3	4.9	-	-

Bold indicate between what groups the largest difference in allele frequencies occurred and where statistics were performed (OR and P-values).
