# Supplementary material for: Multiple Changes of Gene Expression and Function Reveal Genomic and Phenotypic Complexity in SLE-like Disease
Source: PLoS Genet. 2015 Jun 9;11(6):e1005248. doi: 10.1371/journal.pgen.1005248 (PMC4461293; doi:10.1371/journal.pgen.1005248)
Supplement: S4 Table — (PDF) [file pgen.1005248.s011.pdf]

Table S4. Homozygosity at MHC class II observed in the NSDTR population including all genotypes with and without the risk genotype 2.

Genotype	ANA % (n=59)	ANA^{H} % (n=26)	ANA^{S} % (n=27)	Controls % (n=63)	Total population % (n=122)	OR	P-value
Homozygous	71.2	**65.4**	81.5	**27.0**	48.4	5.1	0.0016
Heterozygous	28.8	34.6	18.5	73.0	51.6	-	-

Genotype	ANA % (n=35)	ANA^{H} % (n=24)	ANA^{S} % (n=6)	Controls % (n=54)	Total population % (n=89)	OR	P-value
Homozygous no 2 risk	51.4	**62.5**	16.7	**14.8**	29.2	9.6	<0.0001
Heterozygous no 2 risk	48.6	37.5	83.3	85.2	70.8	-	-

S= Speckled, H=Homogeneous

Bold indicate between what groups the largest difference in allele frequencies occurred and where statistics were performed (OR and P-values).
