# Supplementary material for: Multiple Changes of Gene Expression and Function Reveal Genomic and Phenotypic Complexity in SLE-like Disease
Source: PLoS Genet. 2015 Jun 9;11(6):e1005248. doi: 10.1371/journal.pgen.1005248 (PMC4461293; doi:10.1371/journal.pgen.1005248)
Supplement: S5 Table — (PDF) [file pgen.1005248.s012.pdf]

Table S5. Positions of all SNPs genotyped and p-values for genetic association, minor allele (A1) and major allele (A2) for each phenotype studies.

	ANA-positive (N=52)			ANAH (N=21)			ANAS (N=24)			SRMA (N=66)			ANAH MHC Homozygotes (N=14)			ANAH MHC DQA1*00601 (N=16)			ANAS MHC 2.2 (N=18)		
**Position**	**A1**	**A2**	**P^{raw}**	**A1**	**A2**	**P^{raw}**	**A1**	**A2**	**P^{raw}**	**A1**	**A2**	**P^{raw}**	**A1**	**A2**	**P^{raw}**	**A1**	**A2**	**P^{raw}**	**A1**	**A2**	**P^{raw}**
11_67463150	G	A	0.58	G	A	0.98	G	A	0.23	G	A	0.94	G	A	0.51	G	A	0.50	G	A	0.28
11_67465332	A	C	0.031	A	C	0.55	A	C	0.14	A	C	0.32	A	C	0.44	A	C	0.55	A	C	0.37
11_67479814	A	G	0.32	A	G	0.54	A	G	0.74	A	G	0.38	A	G	0.88	A	G	0.97	A	G	0.95
11_67481323	A	T	0.31	A	T	0.48	A	T	0.78	A	T	0.43	A	T	0.84	A	T	0.86	A	T	0.91
11_67484477	A	G	0.34	A	G	0.50	A	G	0.81	A	G	0.61	A	G	0.82	A	G	0.88	A	G	0.88
11_67485866	A	G	0.00066	A	G	0.077	A	G	0.0050	A	G	0.95	A	G	0.0012	A	G	0.021	A	G	0.11
11_67504858	A	G	0.0052	A	G	0.041	A	G	0.024	A	G	0.92	A	G	0.034	A	G	0.24	A	G	0.054
11_67511882	C	A	0.014	C	A	0.16	C	A	0.074	C	A	0.37	C	A	0.12	C	A	0.24	C	A	0.32
11_67514454	G	A	0.44	G	A	0.45	G	A	0.86	G	A	0.93	G	A	0.90	G	A	0.96	G	A	0.83
11_67516041	G	A	0.0010	G	A	0.041	G	A	0.017	G	A	0.89	G	A	0.034	G	A	0.24	G	A	0.039
11_67517102	G	A	0.61	G	A	0.52	G	A	0.76	G	A	0.56	G	A	0.90	G	A	0.89	G	A	0.68
11_67518063	G	A	0.13	G	A	0.68	G	A	0.22	G	A	0.88	G	A	0.16	G	A	0.48	G	A	0.33
11_67518596	A	G	0.0023	A	G	0.041	A	G	0.054	A	G	0.86	A	G	0.034	A	G	0.24	A	G	0.13
11_67518781	A	G	0.0023	A	G	0.041	A	G	0.054	A	G	0.89	A	G	0.034	A	G	0.24	A	G	0.13
11_67519533	G	A	0.71	NA	NA	NA	G	A	0.34	NA	NA	NA	NA	NA	NA	NA	NA	NA	G	A	0.87
11_67520723	A	T	0.25	A	T	0.72	A	T	0.31	A	T	0.72	A	T	0.19	A	T	0.54	A	T	0.46
11_67523597	C	G	0.87	C	G	0.42	C	G	0.46	NA	NA	NA	C	G	0.66	C	G	0.61	C	G	1
11_67527627	G	A	0.24	G	A	0.70	G	A	0.29	G	A	0.87	G	A	0.21	G	A	0.53	G	A	0.42
11_67531399	A	G	0.44	A	G	0.61	A	G	0.93	A	G	0.30	A	G	0.81	A	G	0.96	A	G	0.74
11_67535953	A	G	0.0012	A	G	0.032	A	G	0.032	A	G	0.98	A	G	0.027	A	G	0.20	A	G	0.082
11_67536642	C	A	0.00032	C	A	0.012	C	A	0.026	C	A	0.90	C	A	0.0100	C	A	0.090	C	A	0.023
11_67536944	A	G	0.0017	A	G	0.041	A	G	0.032	A	G	0.92	A	G	0.051	A	G	0.29	A	G	0.054
11_67537177	G	A	0.00023	G	A	0.041	G	A	0.0018	G	A	0.92	G	A	0.0017	G	A	0.026	G	A	0.039
11_67537363	A	G	0.0013	A	G	0.041	A	G	0.024	A	G	0.98	A	G	0.051	A	G	0.29	A	G	0.039
11_67537493	A	G	0.0020	A	G	0.043	A	G	0.018	A	G	0.91	A	G	0.035	A	G	0.24	A	G	0.040
11_67537924	A	C	0.0033	A	C	0.042	A	C	0.072	A	C	0.98	A	C	0.052	A	C	0.29	A	C	0.13
11_67538032	A	C	0.00079	A	C	0.014	A	C	0.038	A	C	0.90	A	C	0.014	A	C	0.11	A	C	0.099

11_67538806	G	A	0.0010	G	A	0.041	G	A	0.017	G	A	0.95	G	A	0.034	G	A	0.24	G	A	0.039
11_67539578	A	G	0.30	A	G	0.57	A	G	0.38	A	G	0.52	A	G	0.16	A	G	0.62	A	G	0.32
11_67539780	G	A	0.45	G	A	0.62	G	A	0.77	G	A	0.99	G	A	0.14	G	A	0.59	G	A	0.68
11_67539967	G	A	0.34	G	A	0.67	G	A	0.39	G	A	0.47	G	A	0.20	G	A	0.74	G	A	0.33
11_67543652	A	G	0.0016	A	G	0.032	A	G	0.043	A	G	0.98	A	G	0.041	A	G	0.25	A	G	0.082
11_67553409	A	G	0.090	A	G	0.58	A	G	0.42	A	G	0.26	A	G	0.28	A	G	0.36	A	G	0.90
11_67554201	G	A	0.053	G	A	0.57	G	A	0.24	G	A	0.28	G	A	0.22	G	A	0.28	G	A	0.70
11_67557371	C	G	0.074	C	G	0.41	C	G	0.17	C	G	0.97	C	G	0.41	C	G	0.33	C	G	0.29
11_67560132	G	A	0.013	G	A	0.17	G	A	0.32	G	A	0.82	G	A	0.48	G	A	0.32	G	A	0.99
11_67565265	G	A	0.056	G	A	0.31	G	A	0.60	G	A	0.82	G	A	0.82	G	A	0.66	G	A	0.99
11_67570186	A	G	0.27	A	G	0.41	A	G	0.78	A	G	0.35	A	G	0.93	A	G	0.77	A	G	0.87
11_67576318	A	G	0.42	A	G	0.61	A	G	0.91	A	G	0.59	A	G	0.95	A	G	0.75	A	G	0.74
11_67576585	A	G	0.44	A	G	0.83	A	G	0.18	A	G	0.76	A	G	0.84	A	G	0.84	A	G	0.50
11_67577041	G	A	0.39	G	A	0.59	G	A	0.88	G	A	0.56	G	A	0.97	G	A	0.73	G	A	0.76
11_67583114	A	G	0.37	A	G	0.58	A	G	0.86	A	G	0.42	A	G	0.98	A	G	0.72	A	G	0.78
11_67583604	NA	NA	0.43	NA	NA	0.76	NA	NA	0.25	NA	NA	0.54	NA	NA	0.83	NA	NA	0.67	NA	NA	0.28
11_67583635	NA	NA	0.43	NA	NA	0.76	NA	NA	0.25	NA	NA	0.44	NA	NA	0.83	NA	NA	0.67	NA	NA	0.28
24_35972578	C	A	0.026	C	A	0.082	C	A	0.16	C	A	0.89	C	A	0.13	C	A	0.087	C	A	0.36
24_35975474	A	G	0.020	A	G	0.052	A	G	0.19	A	G	0.84	A	G	0.094	A	G	0.061	A	G	0.43
24_35986324	A	G	0.058	A	G	0.25	A	G	0.25	A	G	0.92	A	G	0.77	A	G	0.35	A	G	0.35
24_35990653	NA	NA	0.026	NA	NA	0.13	NA	NA	0.30	NA	NA	0.48	NA	NA	0.21	NA	NA	0.049	NA	NA	0.73
24_35991197	A	C	0.035	A	C	0.22	A	C	0.24	A	C	0.98	A	C	0.45	A	C	0.16	A	C	0.58
24_35996998	A	G	0.017	A	G	0.058	A	G	0.33	A	G	0.88	A	G	0.15	A	G	0.031	A	G	0.73
24_35997849	C	G	0.037	C	G	0.084	C	G	0.51	C	G	0.71	C	G	0.71	C	G	0.27	C	G	0.56
24_36005156	G	A	0.029	G	A	0.16	G	A	0.26	G	A	0.79	G	A	0.38	G	A	0.11	G	A	0.60
24_36011545	G	A	0.0076	G	A	0.090	G	A	0.12	G	A	0.70	G	A	0.21	G	A	0.051	G	A	0.42
24_36026714	T	A	0.052	T	A	0.061	T	A	0.71	T	A	0.91	T	A	0.55	T	A	0.18	T	A	0.74
24_36027202	T	A	0.039	T	A	0.26	T	A	0.33	T	A	0.92	T	A	0.54	T	A	0.20	T	A	0.65
24_36035902	G	A	0.0096	G	A	0.048	G	A	0.099	G	A	0.65	G	A	0.089	G	A	0.058	G	A	0.25
24_36039924	G	A	0.013	G	A	0.042	G	A	0.16	G	A	0.70	G	A	0.081	G	A	0.052	G	A	0.38
24_36052685	G	A	0.22	G	A	0.21	G	A	0.38	G	A	0.28	G	A	0.030	G	A	0.64	G	A	0.75
24_36055061	G	A	0.19	G	A	0.13	G	A	0.41	G	A	0.23	G	A	0.014	G	A	0.45	G	A	0.78

24_36055134	G	A	0.26	G	A	0.22	G	A	0.69	G	A	0.14	G	A	0.030	G	A	0.33	G	A	0.61
24_36058022	A	G	0.0091	A	G	0.015	A	G	0.19	A	G	0.11	A	G	0.0032	A	G	0.076	A	G	0.82
24_36059919	G	C	0.20	G	C	0.21	G	C	0.32	G	C	0.38	G	C	0.020	G	C	0.45	G	C	0.81
24_36060401	A	C	0.32	A	C	0.25	A	C	0.50	A	C	0.32	A	C	0.043	A	C	0.70	A	C	0.89
24_36060972	A	C	0.0065	A	C	0.011	A	C	0.089	A	C	0.34	A	C	0.00096	A	C	0.078	A	C	0.46
24_36063718	A	G	0.0021	A	G	0.0078	A	G	0.037	A	G	0.56	A	G	0.0012	A	G	0.080	A	G	0.28
24_36064479	A	G	0.0057	A	G	0.040	A	G	0.069	A	G	0.63	A	G	0.079	A	G	0.050	A	G	0.18
24_36065249	A	G	0.28	A	G	0.20	A	G	0.55	A	G	0.26	A	G	0.048	A	G	0.68	A	G	0.95
24_36066098	G	A	0.013	G	A	0.0081	G	A	0.22	G	A	0.27	G	A	0.00074	G	A	0.065	G	A	0.81
24_36066521	A	G	0.26	A	G	0.15	A	G	0.58	A	G	0.22	A	G	0.036	A	G	0.46	A	G	0.97
24_36072077	A	G	0.18	A	G	0.18	A	G	0.53	A	G	0.18	A	G	0.035	A	G	0.25	A	G	0.81
24_36073141	A	G	0.013	A	G	0.042	A	G	0.16	A	G	0.70	A	G	0.081	A	G	0.052	A	G	0.38
24_36075020	C	A	0.0067	C	A	0.040	C	A	0.083	C	A	0.68	C	A	0.079	C	A	0.050	C	A	0.22
24_36075761	A	C	0.0048	A	C	0.010	A	C	0.15	A	C	0.21	A	C	0.0024	A	C	0.057	A	C	0.77
24_36087012	C	G	0.0026	C	G	0.0086	C	G	0.079	C	G	0.25	C	G	0.00045	C	G	0.016	C	G	0.71
24_36088520	G	A	0.0029	G	A	0.011	G	A	0.092	G	A	0.36	G	A	0.0025	G	A	0.058	G	A	0.58
24_36093636	A	G	0.36	A	G	0.25	A	G	0.89	A	G	0.23	A	G	0.029	A	G	0.36	A	G	0.41
3_57358881	A	G	0.37	A	G	0.22	A	G	0.73	A	G	0.50	A	G	0.53	A	G	0.13	A	G	0.98
3_57364554	A	G	0.18	A	G	0.43	A	G	0.57	A	G	0.64	A	G	0.37	A	G	0.54	A	G	0.63
3_57377038	A	G	0.00066	A	G	0.0072	A	G	0.049	A	G	0.17	A	G	0.062	A	G	0.033	A	G	0.25
3_57380628	A	G	0.020	A	G	0.067	A	G	0.11	A	G	0.37	A	G	0.21	A	G	0.092	A	G	0.42
3_57387122	A	G	0.075	A	G	0.071	A	G	0.50	A	G	0.47	A	G	0.20	A	G	0.051	A	G	0.78
3_57387381	G	A	0.27	G	A	0.21	G	A	0.78	G	A	0.41	G	A	0.57	G	A	0.15	G	A	0.99
3_57389628	G	A	0.066	G	A	0.065	G	A	0.47	G	A	0.29	G	A	0.19	G	A	0.046	G	A	0.75
3_57391172	A	G	0.18	A	G	0.18	A	G	0.40	A	G	0.31	A	G	0.32	A	G	0.065	A	G	0.73
3_57396626	G	A	0.15	G	A	0.26	G	A	0.53	G	A	0.44	G	A	0.64	G	A	0.75	G	A	0.96
3_57400357	A	G	0.00092	A	G	0.0030	A	G	0.10	A	G	0.17	A	G	0.11	A	G	0.028	A	G	0.38
3_57420873	C	A	0.00058	C	A	0.0044	C	A	0.057	C	A	0.20	C	A	0.012	C	A	0.0065	C	A	0.46
3_57430664	A	T	0.014	A	T	0.17	A	T	0.15	A	T	0.51	A	T	0.16	A	T	0.41	A	T	0.40
3_57432981	T	A	0.00049	T	A	0.0080	T	A	0.033	T	A	0.22	T	A	0.035	T	A	0.018	T	A	0.26
3_57440271	A	G	0.032	A	G	0.20	A	G	0.10	A	G	0.048	A	G	0.15	A	G	0.22	A	G	0.47
3_57441115	A	G	0.020	A	G	0.42	A	G	0.051	A	G	0.041	A	G	0.39	A	G	0.75	A	G	0.16

3_57445901	G	A	0.0055	G	A	0.41	G	A	0.0060	G	A	0.058	G	A	0.16	G	A	0.23	G	A	0.12
3_57450657	A	G	0.022	A	G	0.56	A	G	0.028	A	G	0.064	A	G	0.39	A	G	0.74	A	G	0.18
3_57453525	G	A	0.0069	G	A	0.21	G	A	0.015	G	A	0.031	G	A	0.47	G	A	0.37	G	A	0.085
3_57455143	G	A	0.033	G	A	0.56	G	A	0.063	G	A	0.080	G	A	0.55	G	A	0.66	G	A	0.30
3_57456374	G	A	0.95	G	A	0.38	G	A	0.66	G	A	0.55	G	A	0.27	G	A	0.11	G	A	0.83
3_57457635	A	G	0.26	A	G	0.17	A	G	0.61	A	G	0.42	A	G	0.45	A	G	0.10	A	G	0.86
3_57457738	A	G	0.033	A	G	0.56	A	G	0.089	A	G	0.18	A	G	0.55	A	G	0.66	A	G	0.38
3_57460799	A	G	0.033	A	G	0.57	A	G	0.059	A	G	0.058	A	G	0.68	A	G	0.80	A	G	0.19
3_57464057	G	A	0.017	G	A	0.27	G	A	0.063	G	A	0.053	G	A	0.55	G	A	0.45	G	A	0.30
3_57466015	A	C	0.029	A	C	0.35	A	C	0.10	A	C	0.022	A	C	0.62	A	C	0.73	A	C	0.34
3_57467370	C	A	0.051	C	A	0.39	C	A	0.18	C	A	0.053	C	A	0.66	C	A	0.78	C	A	0.55
3_57482327	G	A	0.012	G	A	0.19	G	A	0.073	G	A	0.17	G	A	0.54	G	A	0.43	G	A	0.28
3_57484486	C	A	0.0056	C	A	0.19	C	A	0.022	C	A	0.027	C	A	0.44	C	A	0.35	C	A	0.13
3_57484658	G	A	0.010	G	A	0.37	G	A	0.021	G	A	0.045	G	A	0.52	G	A	0.64	G	A	0.11
3_57495652	G	A	0.30	G	A	0.18	G	A	0.68	G	A	0.39	G	A	0.47	G	A	0.11	G	A	0.93
3_57500572	A	G	0.019	A	G	0.37	A	G	0.049	A	G	0.068	A	G	0.52	A	G	0.64	A	G	0.23
3_57501076	G	A	0.023	G	A	0.30	G	A	0.29	G	A	0.17	G	A	0.47	G	A	0.45	G	A	0.86
3_57505722	A	G	0.97	A	G	1	NA	NA	NA	A	G	0.90	A	G	0.56	A	G	0.63	NA	NA	NA
3_57510195	G	A	0.012	G	A	0.30	G	A	0.16	G	A	0.12	G	A	0.35	G	A	0.34	G	A	0.72
3_57517286	A	G	0.0095	A	G	0.37	A	G	0.023	A	G	0.037	A	G	0.35	A	G	0.70	A	G	0.13
3_57517383	A	G	0.013	A	G	0.36	A	G	0.032	A	G	0.081	A	G	0.51	A	G	0.62	A	G	0.17
3_57517805	G	A	0.24	G	A	0.76	G	A	0.18	G	A	0.27	G	A	0.85	G	A	0.87	G	A	0.42
3_57520708	A	G	0.37	A	G	0.88	A	G	0.27	A	G	0.33	A	G	0.67	A	G	0.81	A	G	0.44
3_57521471	A	G	0.026	A	G	0.21	A	G	0.063	A	G	0.17	A	G	0.47	A	G	0.37	A	G	0.31
3_57521716	G	A	0.38	G	A	0.82	G	A	0.37	G	A	0.33	G	A	0.72	G	A	0.86	G	A	0.62
3_57526312	G	A	0.22	G	A	0.71	G	A	0.17	G	A	0.16	G	A	0.98	G	A	0.87	G	A	0.44
3_57527697	G	A	0.23	G	A	0.60	G	A	0.27	G	A	0.096	G	A	0.78	G	A	0.66	G	A	0.32
3_57537169	A	G	0.93	A	G	0.52	A	G	0.64	A	G	0.63	A	G	0.29	A	G	0.19	A	G	0.81
3_57540129	A	G	0.74	A	G	0.53	A	G	0.42	A	G	0.50	A	G	0.43	A	G	0.30	A	G	0.65
3_57543028	C	A	0.11	C	A	0.33	C	A	0.45	C	A	0.94	C	A	0.30	C	A	0.45	C	A	0.52
3_57546568	G	A	0.040	G	A	0.019	G	A	0.33	G	A	0.96	G	A	0.0033	G	A	0.0012	G	A	0.77
3_57547350	G	A	0.94	G	A	0.41	G	A	0.72	G	A	0.55	G	A	0.29	G	A	0.12	G	A	0.92

3_57553803	G	A	0.11	G	A	0.35	G	A	0.41	G	A	0.75	G	A	0.26	G	A	0.40	G	A	0.55
3_57556367	A	G	0.22	A	G	0.98	A	G	0.29	A	G	0.76	A	G	0.90	A	G	0.36	A	G	0.45
3_57556909	G	A	0.33	G	A	0.85	G	A	0.38	G	A	0.75	G	A	0.79	G	A	0.30	G	A	0.57
3_57563821	A	G	0.87	A	G	0.42	A	G	0.43	A	G	0.86	A	G	0.35	A	G	0.23	A	G	0.64
3_57563964	G	A	0.23	G	A	0.90	G	A	0.25	G	A	0.73	G	A	0.82	G	A	0.33	G	A	0.38
3_57564331	G	A	0.82	G	A	0.32	G	A	0.76	G	A	0.55	G	A	0.27	G	A	0.087	G	A	0.95
3_57565870	A	G	0.35	A	G	0.63	A	G	0.27	A	G	0.72	A	G	0.54	A	G	0.17	A	G	0.40
3_57569227	G	A	0.46	G	A	0.86	G	A	0.62	G	A	0.79	G	A	0.51	G	A	0.17	G	A	0.60
3_57569427	G	A	0.16	G	A	0.71	G	A	0.20	G	A	0.91	G	A	0.76	G	A	0.64	G	A	0.32
3_57570790	G	A	0.34	G	A	0.86	G	A	0.41	G	A	0.66	G	A	0.80	G	A	0.31	G	A	0.60
3_57575243	G	A	0.0019	G	A	0.044	G	A	0.026	G	A	0.23	G	A	0.47	G	A	0.18	G	A	0.14
3_57584616	G	A	0.052	G	A	0.078	G	A	0.35	G	A	0.65	G	A	0.14	G	A	0.071	G	A	0.62
3_57587513	A	G	0.035	A	G	0.041	A	G	0.36	A	G	0.84	A	G	0.14	A	G	0.031	A	G	0.62
3_57591964	A	G	0.037	A	G	0.043	A	G	0.37	A	G	0.76	A	G	0.14	A	G	0.032	A	G	0.64
3_57601647	C	G	0.59	NA	NA	NA	NA	NA	NA	NA	NA	NA	NA	NA	NA	NA	NA	NA	NA	NA	NA
32_24524629	G	A	0.023	G	A	0.30	G	A	0.035	G	A	0.72	G	A	0.10	G	A	0.19	G	A	0.0045
32_24534623	C	G	0.016	C	G	0.26	C	G	0.027	C	G	0.38	C	G	0.086	C	G	0.16	C	G	0.0032
32_24534923	A	G	0.029	A	G	0.39	A	G	0.043	A	G	0.78	A	G	0.069	A	G	0.14	A	G	0.0059
32_24535749	G	A	0.033	G	A	0.29	G	A	0.067	G	A	0.74	G	A	0.10	G	A	0.19	G	A	0.010
32_24536306	A	T	0.030	A	T	0.34	A	T	0.044	A	T	0.65	A	T	0.12	A	T	0.22	A	T	0.0060
32_24542001	A	G	0.0051	A	G	0.26	A	G	0.0040	A	G	0.49	A	G	0.0074	A	G	0.021	A	G	0.0032
32_24553050	G	A	0.024	G	A	0.25	G	A	0.053	G	A	0.64	G	A	0.083	G	A	0.16	G	A	0.0077
32_24556037	G	A	0.013	G	A	0.97	G	A	0.026	G	A	0.28	G	A	0.55	G	A	0.66	G	A	0.018
32_24556482	A	G	0.30	A	G	0.24	NA	NA	NA	A	G	0.90	A	G	0.56	NA	NA	NA	A	G	0.89
32_24565468	G	A	0.020	G	A	0.26	G	A	0.037	G	A	0.77	G	A	0.045	G	A	0.067	G	A	0.012
32_24577310	G	A	0.54	G	A	0.40	G	A	0.65	G	A	0.54	G	A	0.74	G	A	0.54	G	A	0.90
32_24577939	A	G	0.097	A	G	0.41	A	G	0.37	A	G	0.0065	A	G	0.54	A	G	0.85	A	G	0.12
32_24578855	NA	NA	NA	NA	NA	NA	NA	NA	NA	A	G	0.093	NA	NA	NA	NA	NA	NA	NA	NA	NA
32_24580618	A	G	0.77	A	G	0.30	A	G	0.82	A	G	0.79	A	G	0.60	A	G	0.21	A	G	0.55
32_24585861	A	G	0.49	A	G	0.13	A	G	1.00	A	G	0.36	A	G	0.38	A	G	0.020	A	G	0.58
32_24586843	G	C	0.62	G	C	0.24	G	C	0.76	G	C	0.24	G	C	0.27	G	C	0.068	G	C	0.33
32_24594944	A	G	0.32	A	G	0.39	A	G	0.79	A	G	0.15	A	G	0.47	A	G	0.045	G	A	0.35

32_24596172	A	G	0.10	A	G	0.53	A	G	0.22	A	G	0.0051	A	G	0.96	A	G	0.40	A	G	0.20
32_24606503	A	G	0.47	A	G	0.11	A	G	0.45	A	G	0.46	A	G	0.20	A	G	0.18	A	G	0.97
32_24618331	C	G	0.63	C	G	0.091	C	G	0.57	C	G	0.64	C	G	0.18	C	G	0.15	C	G	0.84
32_24650093	A	G	0.98	A	G	0.10	A	G	0.85	A	G	0.38	A	G	0.20	A	G	0.17	A	G	0.97
32_24667283	A	G	0.88	NA	NA	NA	A	G	0.81	A	G	0.35	NA	NA	NA	NA	NA	NA	A	G	0.97
32_24672221	A	G	0.95	A	G	0.10	A	G	0.90	A	G	0.65	A	G	0.20	A	G	0.17	A	G	0.90
32_24672306	A	G	0.29	A	G	0.84	A	G	0.44	A	G	0.0072	A	G	0.85	A	G	0.16	A	G	0.61
32_24688953	A	G	0.91	G	A	0.52	A	G	0.82	G	A	0.089	G	A	0.45	G	A	0.067	A	G	0.35
32_24690295	G	C	0.89	G	C	0.48	G	C	0.70	C	G	0.043	G	C	0.91	C	G	0.26	G	C	0.62
32_24741236	G	A	0.29	G	A	0.95	G	A	0.39	G	A	0.46	G	A	0.75	G	A	0.43	G	A	0.61
32_24744042	A	G	0.51	A	G	0.51	A	G	0.23	A	G	0.68	A	G	0.31	A	G	0.51	A	G	0.063
32_24746997	A	G	0.38	A	G	0.63	A	G	0.21	A	G	0.032	A	G	0.90	A	G	0.74	A	G	0.27
32_24751017	A	G	0.59	A	G	0.32	A	G	0.24	A	G	0.024	A	G	0.65	A	G	0.85	A	G	0.31
32_24815472	C	G	0.27	C	G	0.96	C	G	0.27	C	G	0.0043	C	G	0.97	C	G	0.99	C	G	0.25
32_24827518	A	G	0.77	A	G	0.51	A	G	0.71	A	G	0.00055	A	G	0.94	A	G	0.90	A	G	0.95
32_24854147	G	A	0.65	G	A	0.88	G	A	0.80	G	A	0.018	G	A	0.65	G	A	0.78	G	A	0.79
32_24872857	A	G	0.76	A	G	0.67	A	G	0.69	G	A	0.075	A	G	0.39	A	G	0.42	A	G	0.42
32_24884985	C	G	0.84	C	G	0.34	C	G	0.58	C	G	0.010	C	G	0.34	C	G	0.37	C	G	0.37
32_24890208	A	G	0.92	A	G	0.47	A	G	0.66	A	G	0.0048	A	G	0.44	A	G	0.50	A	G	0.48
32_24985562	G	A	0.50	G	A	0.37	G	A	0.75	G	A	0.0043	G	A	0.89	G	A	0.55	G	A	0.40
32_24987404	G	A	0.99	G	A	0.97	G	A	0.84	G	A	0.0015	G	A	0.90	G	A	0.97	G	A	0.59
32_24987634	G	A	0.72	G	A	0.70	G	A	0.77	G	A	0.0055	G	A	0.86	G	A	0.99	G	A	0.42
32_24991562	C	A	0.22	C	A	0.81	C	A	0.072	C	A	0.52	C	A	0.48	C	A	0.50	C	A	0.0087
32_25007496	G	A	0.13	G	A	0.57	G	A	0.098	G	A	0.53	G	A	0.68	G	A	0.57	G	A	0.063
32_25007632	C	A	0.084	C	A	0.66	C	A	0.098	C	A	0.53	C	A	0.81	C	A	0.68	C	A	0.063
32_25045020	A	G	0.41	A	G	0.46	A	G	0.55	A	G	0.012	A	G	0.63	A	G	0.61	A	G	0.37
32_25049586	A	G	0.84	A	G	0.091	A	G	0.98	A	G	0.61	A	G	0.18	A	G	0.15	A	G	0.84
32_25051669	A	C	1	A	C	1	A	C	0.79	C	A	0.076	C	A	0.45	C	A	0.47	A	C	0.35
32_25087394	A	C	0.60	A	C	0.94	A	C	0.87	A	C	0.017	A	C	0.97	A	C	0.99	A	C	0.74
32_25155250	G	A	0.72	G	A	0.38	G	A	0.43	G	A	0.073	G	A	0.22	G	A	0.22	G	A	0.16
32_25160803	A	G	0.047	A	G	0.73	A	G	0.037	A	G	0.45	A	G	0.73	A	G	0.85	A	G	0.011

32_25192809	C	A	0.35	C	A	0.42	C	A	0.067	C	A	0.99	C	A	0.54	C	A	0.44	C	A	0.013
32_25220606	A	G	0.84	A	G	0.33	A	G	0.39	A	G	0.0069	A	G	0.79	A	G	0.65	A	G	0.45
32_25244162	A	G	0.74	A	G	0.37	A	G	0.46	A	G	0.13	A	G	0.21	A	G	0.22	A	G	0.19
32_25244721	G	A	0.88	G	A	0.28	G	A	0.55	G	A	0.097	G	A	0.25	G	A	0.25	G	A	0.25
32_25258608	A	T	0.049	A	T	0.62	A	T	0.053	A	T	0.49	A	T	0.68	A	T	0.68	A	T	0.017
32_25298105	G	A	0.20	G	A	0.14	G	A	0.27	G	A	0.72	G	A	0.11	G	A	0.092	G	A	0.18
32_25305524	G	A	0.49	G	A	0.042	G	A	0.93	G	A	0.44	G	A	0.11	G	A	0.085	G	A	0.88
32_25309417	T	A	0.19	T	A	0.11	T	A	0.51	T	A	0.30	T	A	0.15	T	A	0.11	T	A	0.71
32_25310777	C	A	0.20	C	A	0.12	C	A	0.51	C	A	0.088	C	A	0.16	C	A	0.13	C	A	0.70
32_25312137	C	A	0.58	C	A	0.60	C	A	0.53	C	A	0.35	C	A	0.35	C	A	0.36	C	A	0.26
32_25339008	A	T	0.52	A	T	0.86	A	T	0.62	A	T	0.44	A	T	0.51	A	T	0.80	A	T	0.36
32_25363099	G	A	0.34	G	A	0.88	G	A	0.21	G	A	0.0025	G	A	0.78	G	A	0.77	G	A	0.31
32_25392401	A	G	0.29	A	G	0.24	A	G	0.084	A	G	0.087	A	G	0.36	A	G	0.28	A	G	0.033
32_25444674	G	A	0.63	G	A	0.50	G	A	0.91	G	A	0.42	G	A	0.17	G	A	0.13	G	A	0.62
32_25473375	NA	NA	NA	NA	NA	NA	A	T	0.16	A	T	0.11	NA	NA	NA	NA	NA	NA	A	T	0.055
32_25473551	A	G	0.65	A	G	0.99	A	G	0.48	A	G	0.31	A	G	0.52	A	G	0.43	A	G	0.72
32_25473649	G	A	0.46	G	A	0.38	G	A	0.94	G	A	0.74	G	A	0.22	G	A	0.16	G	A	0.62
32_25484844	A	G	0.23	A	G	0.54	A	G	0.17	A	G	0.039	A	G	0.19	A	G	0.13	A	G	0.034
32_25485644	A	G	0.081	A	G	0.86	A	G	0.14	A	G	0.066	A	G	0.76	A	G	0.54	A	G	0.11
32_25485961	A	G	0.086	A	G	0.90	A	G	0.17	A	G	0.074	A	G	0.60	A	G	0.50	A	G	0.083
32_25512953	T	A	0.28	T	A	0.58	T	A	0.48	T	A	0.00060	T	A	0.80	T	A	0.81	T	A	0.54
32_25537276	G	A	0.19	G	A	0.62	G	A	0.082	G	A	0.036	G	A	0.47	G	A	0.47	G	A	0.032
32_25575042	T	A	0.029	T	A	0.28	T	A	0.21	T	A	0.25	T	A	0.66	T	A	0.40	T	A	0.093
32_25584449	A	G	0.26	A	G	0.81	A	G	0.23	A	G	0.0028	A	G	1.00	A	G	0.98	A	G	0.21
32_25584847	A	C	0.20	A	C	0.88	A	C	0.19	A	C	0.0021	A	C	0.94	A	C	0.91	A	C	0.15
32_25600886	C	A	0.45	C	A	0.11	C	A	0.81	C	A	0.33	C	A	0.045	C	A	0.026	C	A	0.49
32_25608310	A	C	0.75	A	C	0.36	A	C	0.45	A	C	0.49	A	C	0.20	A	C	0.21	A	C	0.17
32_25611054	A	G	0.77	A	G	0.36	A	G	0.48	A	G	0.49	A	G	0.20	A	G	0.21	A	G	0.20
32_25611296	A	G	0.14	A	G	0.015	A	G	0.76	A	G	0.29	A	G	0.057	A	G	0.047	A	G	0.79
32_25614460	C	A	0.50	C	A	0.11	C	A	0.76	C	A	0.43	C	A	0.045	C	A	0.027	C	A	0.47
32_25614950	G	A	0.44	G	A	0.099	G	A	0.81	G	A	0.47	G	A	0.041	G	A	0.024	G	A	0.51

32_25615766	A	G	0.58	A	G	0.40	A	G	0.29	A	G	0.80	A	G	0.41	A	G	0.41	A	G	0.15
32_25642357	T	A	0.98	T	A	0.086	T	A	0.67	T	A	0.16	T	A	0.17	T	A	0.15	T	A	0.76
32_25662759	A	G	0.72	A	G	0.17	A	G	0.79	A	G	0.42	A	G	0.088	A	G	0.057	A	G	0.64
32_25662984	A	G	0.38	A	G	0.44	A	G	0.28	A	G	0.17	A	G	0.16	A	G	0.16	A	G	0.094
32_25701735	A	G	0.23	A	G	0.90	A	G	0.38	A	G	0.43	A	G	0.86	A	G	0.56	A	G	0.48
32_25702963	G	A	0.90	G	A	0.097	G	A	0.93	G	A	0.13	G	A	0.18	G	A	0.16	G	A	0.89
32_25710678	C	A	0.095	C	A	0.86	C	A	0.23	C	A	0.20	C	A	0.44	C	A	0.59	C	A	0.11
32_25714903	A	G	0.47	A	G	0.98	A	G	0.61	A	G	0.0028	A	G	0.84	A	G	0.81	A	G	0.78
32_25718852	G	A	0.32	G	A	0.0054	G	A	0.54	G	A	0.23	G	A	0.028	G	A	0.018	G	A	0.34
32_25726018	A	G	0.27	A	G	0.90	A	G	0.34	A	G	0.0056	A	G	0.93	A	G	0.90	A	G	0.31
32_25736276	A	G	0.47	A	G	0.93	A	G	0.47	A	G	1.00	A	G	0.41	A	G	0.66	A	G	0.24
32_25738635	G	A	0.29	G	A	0.58	G	A	0.17	G	A	0.011	G	A	1.00	G	A	0.98	G	A	0.19
32_25764499	A	G	0.18	A	G	0.74	A	G	0.23	A	G	0.16	A	G	0.80	A	G	0.80	A	G	0.068
32_25779083	C	A	0.57	C	A	0.045	C	A	0.66	C	A	0.54	C	A	0.11	C	A	0.090	C	A	0.66
32_25792975	G	A	0.063	G	A	0.98	G	A	0.038	G	A	0.060	G	A	0.94	G	A	0.94	G	A	0.0077
32_25798353	G	A	0.048	G	A	0.72	G	A	0.017	G	A	0.047	G	A	0.56	G	A	0.97	G	A	0.0071
32_25816401	A	G	0.44	A	G	0.20	A	G	0.18	A	G	0.16	A	G	0.16	A	G	0.16	A	G	0.048
32_25829058	A	T	0.019	A	T	0.35	A	T	0.083	A	T	0.53	A	T	0.75	A	T	0.49	A	T	0.024
32_25851447	G	A	0.49	G	A	0.73	G	A	0.50	G	A	0.16	G	A	0.62	G	A	0.77	G	A	0.49
32_25852721	A	G	0.39	A	G	0.96	A	G	0.65	A	G	0.37	A	G	0.72	A	G	0.93	A	G	0.56
32_25856481	NA	NA	NA	NA	NA	NA	NA	NA	NA	A	G	0.15	NA	NA	NA	NA	NA	NA	NA	NA	NA
32_25861294	A	G	0.69	A	G	0.46	A	G	0.97	A	G	0.26	A	G	0.52	A	G	0.63	A	G	0.74
32_25874718	A	G	0.096	A	G	0.96	A	G	0.079	A	G	0.084	A	G	0.82	A	G	0.93	A	G	0.019
32_25896361	T	A	0.057	T	A	0.77	T	A	0.052	T	A	0.068	T	A	0.70	T	A	0.82	T	A	0.0071
32_25907124	A	G	0.034	A	G	0.35	A	G	0.18	A	G	0.28	A	G	0.75	A	G	0.49	A	G	0.069
32_25908297	A	C	0.40	A	C	0.81	A	C	0.28	A	C	0.34	A	C	0.64	A	C	0.53	A	C	0.41
32_25914869	G	A	0.17	G	A	0.83	G	A	0.19	G	A	0.19	G	A	0.50	G	A	0.67	G	A	0.026
32_25918398	C	G	0.28	C	G	0.97	C	G	0.30	C	G	0.010	C	G	0.91	C	G	0.74	C	G	0.29
32_25919171	T	A	0.22	T	A	0.65	T	A	0.29	T	A	0.65	T	A	0.98	T	A	0.76	T	A	0.21
32_25919861	C	G	0.36	C	G	0.44	C	G	0.078	C	G	0.29	C	G	0.58	C	G	0.48	C	G	0.13
32_25948216	C	G	0.46	C	G	0.79	C	G	0.46	C	G	0.092	C	G	0.95	C	G	0.60	C	G	0.28
32_25952535	C	G	0.27	C	G	0.78	C	G	0.19	C	G	0.11	C	G	0.59	C	G	0.34	C	G	0.17

32_25954493	A	T	0.36	A	T	0.80	A	T	0.30	A	T	0.057	A	T	0.61	A	T	0.35	A	T	0.28
32_25969707	G	A	0.091	G	A	0.86	G	A	0.14	G	A	0.58	G	A	0.93	G	A	0.93	G	A	0.052
32_25999207	A	C	0.63	A	C	0.14	A	C	0.35	A	C	0.20	A	C	0.34	A	C	0.34	A	C	0.33
32_26024845	C	A	0.34	C	A	0.088	C	A	0.59	C	A	0.32	C	A	0.18	C	A	0.15	C	A	0.32
32_26048552	A	G	0.12	A	G	0.073	A	G	0.81	A	G	0.48	A	G	0.14	A	G	0.12	A	G	0.81
32_26075658	A	G	0.75	A	G	0.24	A	G	0.30	A	G	0.64	A	G	0.30	A	G	0.30	A	G	0.085
32_26115349	A	T	0.28	A	T	0.89	A	T	0.38	A	T	0.0007<br>0	A	T	0.91	A	T	0.95	A	T	0.44
32_26124413	G	A	0.61	G	A	0.55	G	A	0.31	G	A	0.62	G	A	0.22	G	A	0.39	G	A	0.099
32_26137366	G	A	0.043	G	A	0.017	G	A	0.87	G	A	0.93	G	A	0.059	G	A	0.043	G	A	0.68
32_26146413	G	A	0.42	G	A	0.10	G	A	0.84	G	A	0.23	G	A	0.043	G	A	0.025	G	A	0.52
32_26169472	G	A	0.22	G	A	0.61	G	A	0.42	G	A	0.42	G	A	0.74	G	A	0.92	G	A	0.27
32_26172686	G	A	0.88	G	A	0.29	G	A	0.59	G	A	0.24	G	A	0.10	G	A	0.21	G	A	0.22
32_26192058	A	C	0.66	A	C	0.85	A	C	0.48	A	C	0.043	A	C	0.73	A	C	0.84	A	C	0.47
32_26216458	A	G	0.62	A	G	0.78	A	G	0.78	A	G	0.016	A	G	0.42	A	G	0.51	A	G	0.87
32_26216852	A	T	0.65	A	T	0.52	A	T	0.33	A	T	0.81	A	T	0.20	A	T	0.37	A	T	0.11
32_26242581	G	A	0.28	G	A	0.50	G	A	0.42	G	A	0.090	G	A	0.73	G	A	0.37	G	A	0.27
32_26265151	C	A	0.28	C	A	0.64	C	A	0.43	C	A	0.34	C	A	0.44	C	A	0.51	C	A	0.64
32_26273290	A	G	0.19	A	G	0.26	A	G	0.58	A	G	0.087	A	G	0.19	A	G	0.15	A	G	0.77
32_26299528	C	A	0.21	C	A	0.11	C	A	0.43	C	A	0.28	C	A	0.23	C	A	0.12	C	A	0.97
32_26300452	G	A	0.45	G	A	0.46	G	A	0.34	G	A	0.067	G	A	0.40	G	A	0.35	G	A	0.24
8_68691252	NA	NA	NA	NA	NA	NA	G	C	0.74	NA	NA	NA	NA	NA	NA	NA	NA	NA	G	C	0.95
8_68692173	NA	NA	NA	NA	NA	NA	G	A	0.82	NA	NA	NA	G	A	0.21	G	A	0.18	G	A	0.98
8_68693211	NA	NA	NA	NA	NA	NA	G	A	0.88	NA	NA	NA	G	A	0.20	G	A	0.17	G	A	0.98
8_68693687	NA	NA	NA	NA	NA	NA	A	C	0.82	NA	NA	NA	A	C	0.23	A	C	0.20	A	C	0.98
8_68695255	NA	NA	NA	NA	NA	NA	G	A	0.75	NA	NA	NA	NA	NA	NA	NA	NA	NA	G	A	0.96
8_68697095	G	A	0.46	NA	NA	NA	G	A	0.49	NA	NA	NA	G	A	0.64	G	A	0.54	G	A	0.97
8_68697840	G	A	0.31	G	A	0.37	G	A	0.53	G	A	0.75	G	A	0.79	G	A	0.57	G	A	0.41
8_68700154	A	G	0.27	A	G	0.26	A	G	0.57	A	G	0.78	A	G	0.83	A	G	0.39	A	G	0.43
8_68704546	A	G	0.94	A	G	0.30	A	G	0.78	A	G	0.28	A	G	0.38	A	G	0.21	A	G	0.87
8_68706010	G	A	0.18	G	A	0.75	G	A	0.14	G	A	0.41	G	A	0.52	G	A	0.40	G	A	0.15
8_68707064	G	A	0.18	G	A	0.45	G	A	0.28	G	A	0.081	G	A	0.49	G	A	0.74	G	A	0.36

8_68708204	A	G	0.19	A	G	0.033	A	G	0.68	A	G	0.084	A	G	0.41	A	G	0.033	A	G	0.48
8_68708503	A	C	0.044	A	C	0.019	NA	NA	NA	NA	NA	NA	A	C	0.064	A	C	0.0087	NA	NA	NA
8_68710009	G	A	0.23	G	A	0.34	G	A	0.65	G	A	0.90	G	A	0.18	G	A	0.53	G	A	0.85
8_68712185	A	G	0.14	A	G	0.028	A	G	0.51	A	G	0.053	A	G	0.098	A	G	0.0067	A	G	0.62
8_68714106	A	G	0.19	A	G	0.35	A	G	0.51	A	G	0.96	A	G	0.19	A	G	0.55	A	G	0.67
8_68715568	A	G	0.23	A	G	0.34	A	G	0.65	A	G	0.85	A	G	0.18	A	G	0.53	A	G	0.85
8_68719325	C	A	0.19	C	A	0.45	C	A	0.38	C	A	0.82	C	A	0.24	C	A	0.65	C	A	0.79
8_68721904	A	G	0.16	A	G	0.43	A	G	0.32	A	G	0.57	A	G	0.23	A	G	0.64	A	G	0.67
8_68723075	C	A	0.17	C	A	0.42	C	A	0.36	C	A	0.87	C	A	0.23	C	A	0.63	C	A	0.76
8_68726546	A	G	0.26	A	G	0.54	A	G	0.21	A	G	0.0022	A	G	0.56	A	G	0.17	A	G	0.14
8_68732160	A	G	0.94	A	G	0.29	A	G	0.80	A	G	0.061	A	G	0.81	A	G	0.13	A	G	0.70
8_68732728	A	G	0.76	A	G	0.29	A	G	0.38	A	G	0.061	A	G	0.64	A	G	0.22	A	G	0.90
8_68733351	NA	NA	0.89	NA	NA	0.39	NA	NA	0.67	NA	NA	0.028	NA	NA	0.81	NA	NA	0.22	NA	NA	0.86
8_68733356	NA	NA	0.83	NA	NA	0.39	NA	NA	0.57	NA	NA	0.028	NA	NA	0.81	NA	NA	0.22	NA	NA	0.98
8_68734829	G	A	0.55	G	A	0.10	G	A	0.95	G	A	0.096	G	A	1	G	A	0.097	G	A	0.68
8_68740341	A	G	0.65	A	G	0.11	A	G	0.78	A	G	0.11	A	G	0.99	A	G	0.10	A	G	0.87
8_68742377	A	G	0.55	A	G	0.087	A	G	0.86	A	G	0.095	A	G	0.85	A	G	0.15	A	G	0.62
8_68745098	A	G	0.73	A	G	0.11	A	G	0.63	A	G	0.080	A	G	0.99	A	G	0.10	A	G	0.97
8_68745772	T	A	0.56	T	A	0.11	T	A	0.93	T	A	0.069	T	A	0.99	T	A	0.10	T	A	0.69
8_68747073	G	A	0.075	G	A	0.036	G	A	0.35	G	A	0.75	G	A	0.54	G	A	0.088	G	A	0.35
8_68753958	A	G	0.064	A	G	0.031	A	G	0.33	A	G	0.73	A	G	0.51	A	G	0.079	A	G	0.33
8_68760031	G	A	0.29	G	A	0.20	G	A	0.71	G	A	0.86	G	A	0.62	G	A	0.69	G	A	0.88
8_68770114	G	A	0.79	G	A	0.15	G	A	0.92	G	A	0.073	G	A	0.80	G	A	0.38	G	A	0.76
8_68775560	C	A	0.97	C	A	0.20	C	A	0.79	C	A	0.022	C	A	0.90	C	A	0.45	C	A	0.64
8_68775697	G	C	0.74	G	C	0.16	G	C	0.98	G	C	0.067	G	C	0.81	G	C	0.39	G	C	0.87
8_68780048	A	G	0.62	A	G	0.19	A	G	0.28	A	G	0.041	A	G	0.69	A	G	0.82	A	G	0.57
8_68785200	G	A	0.39	G	A	0.10	G	A	0.85	G	A	0.059	G	A	0.36	G	A	0.18	G	A	0.91
