# Supplementary material for: Multiple Changes of Gene Expression and Function Reveal Genomic and Phenotypic Complexity in SLE-like Disease
Source: PLoS Genet. 2015 Jun 9;11(6):e1005248. doi: 10.1371/journal.pgen.1005248 (PMC4461293; doi:10.1371/journal.pgen.1005248)
Supplement: S6 Table — (PDF) [file pgen.1005248.s013.pdf]

Table S6. Association of differential expression of *WFDC3* with genotyped variants on chromosome 24.

Genes	***WFDC3***
**SNPs genotyped**	gene expression median-fold change, P-value*
36011545	ND
36063718	1.4X, *P=0.0061*
36066098	1.4X, *P=0.0076*
36075020	ND
36075761	1.4X, *P=0.0039*
36082324	1.4X, *P=0.0061*
**36087012**	1.4X, *P=0.0075*

* Correlation was performed by ANOVA, ND – no difference
