# Supplementary material for: Multiple Changes of Gene Expression and Function Reveal Genomic and Phenotypic Complexity in SLE-like Disease
Source: PLoS Genet. 2015 Jun 9;11(6):e1005248. doi: 10.1371/journal.pgen.1005248 (PMC4461293; doi:10.1371/journal.pgen.1005248)
Supplement: S7 Table — (PDF) [file pgen.1005248.s014.pdf]

Table S7. Association of differential expression of genes with genotyped variants across the chromosome 32 locus.

Genes	*DAPP1*	*LAMTOR3*	*DDIT4L*	*PPP3CA*	*BANK1*
**SNPs genotyped**	gene expression median-fold change, P-value*	gene expression median-fold change, P-value*	gene expression median-fold change, P-value*	gene expression median-fold change, P-value*	gene expression median-fold change, P-value*
**24542001**	ND	ND	ND	ND	1.5X, P=0.0007
24556037	ND	ND	2.3X, *P=0.0038*	ND	1.25X, P=0.0027
24606503	ND	ND	ND	ND	ND
24667283	ND	ND	ND	ND	ND
24667774	ND	ND	ND	ND	ND
24672221	ND	ND	ND	ND	ND
**24827518**	1.7X, *P=0.0012*	2X, *P=0.0076*	2.5X, *P<0.0001*	2X, *P=0.0133*	ND
24890208	1.9X, *P=0.049*	1.25X, *P=0.0594*	2X, *P=0.0005*	ND	1.2X, P=0.0172
24985562	2X, *P=0.0233*	ND	2.5X, *P=0.0068*	ND	1.2X, P=0.0042
24987404	1.9X, *P=0.0928*	ND	2.3X, *P=0.0256*	ND	1.2X, P=0.0078
25007496	ND	ND	1.6X, *P=0.0638*	ND	ND
25305524	ND	ND	ND	ND	ND
25363099	ND	ND	ND	ND	ND
25485961	ND	ND	2.3X, *P=0.0001*	ND	1.2X, P=0.0045
25512953	2X, *P=0.0637*	1.7X, *P=0.0231*	1.9X, *P=0.0532*	1.8X, *P=0.0336*	ND
25537276	ND	ND	ND	ND	1.2X, p=0.0321
25537876	ND	ND	ND	ND	1.2X, P=0.0434
25642357	ND	ND	ND	ND	ND
25702963	ND	ND	ND	ND	ND
25714903	ND	1.9X, *P=0.0755*	1.8X, *P=0.0233*	1.8X, *P=0.0264*	ND
25718852	ND	ND	ND	ND	ND
25779083	ND	ND	ND	ND	ND
25798353	ND	ND	ND	ND	1.17X, p=0.0455
26115349	ND	2X, *P=0.0719*	1.5X, *P=0.0345*	1.7X, *P=0.0247*	ND

* Correlation was performed by ANOVA, ND – no difference
