# Supplementary material for: Multiple Changes of Gene Expression and Function Reveal Genomic and Phenotypic Complexity in SLE-like Disease
Source: PLoS Genet. 2015 Jun 9;11(6):e1005248. doi: 10.1371/journal.pgen.1005248 (PMC4461293; doi:10.1371/journal.pgen.1005248)
Supplement: S8 Table — (PDF) [file pgen.1005248.s015.pdf]

Table S8. Association of differential expression of genes with genotyped variants on chromosome 3.

**Genes**	***AP3B2***	***WHAMM***	***HOMER2***
**SNPs genotyped**	gene expression median-fold change, P-value*	gene expression median-fold change, P-value*	gene expression median-fold change, P-value*
57377038	1.3X, *P=0.0112*	1.4X, *P=0.0001*	ND
57400357	1.1X, *P=0.0212*	1.4X, *P=0.0003*	ND
57420873	1.1X, *P=0.0223*	1.4X, *P<0.0001*	ND
**57432981**	1.07X, *P=0.0356*	1.4X, *P<0.0001*	ND
57441115	ND	ND	ND
57457738	ND	ND	ND
57466015	ND	ND	ND
57484486	1.5X, *P=0.0036*	ND	ND
57484658	ND	ND	ND
57500572	ND	ND	ND
57517383	ND	ND	ND
57526312	ND	1.25X; *P=0.0004*	ND
57527697	1.6X, *P=0.0186*	1.2X, *P=0.012*	ND
57546568	ND	1.4X, *P=0.0046*	ND, Thr->Ala
57564331	ND	1.26X, *P<0.0001*	1.4X, P=0.0924
57575243	ND	ND	ND
57610712	ND	1.3X, *P<0.0001*	ND

* Correlation was performed by ANOVA, ND – no difference
