# Supplementary material for: Multiple Changes of Gene Expression and Function Reveal Genomic and Phenotypic Complexity in SLE-like Disease
Source: PLoS Genet. 2015 Jun 9;11(6):e1005248. doi: 10.1371/journal.pgen.1005248 (PMC4461293; doi:10.1371/journal.pgen.1005248)
Supplement: S9 Table — (PDF) [file pgen.1005248.s016.pdf]

Table S9. Clinical manifestations in ANA positive NSDTRs.

clinical signs	all ANA positive	ANA^H	ANA^S
stiffness	100%		
joint pain	100%		
skin lesions	13%	40%	-
muscle pain	25%	20%	27%
fever	13%	-	13%

Complete clinical data were available for 25 NSDTRs collected in Uppsala and examined at the University Animal Hospital at the Swedish University of Agricultural Sciences (SUAS). The remaining 27 dogs collected elsewhere else were diagnozed by local veterinarians as having IMRD, ANA pattern for all dogs was assayed in Uppsala.
