# Supplementary material for: Multiple Changes of Gene Expression and Function Reveal Genomic and Phenotypic Complexity in SLE-like Disease
Source: PLoS Genet. 2015 Jun 9;11(6):e1005248. doi: 10.1371/journal.pgen.1005248 (PMC4461293; doi:10.1371/journal.pgen.1005248)
Supplement: S11 Table — (PDF) [file pgen.1005248.s018.pdf]

Table S11. Primer pairs used for genotyping in expression samples.

CFA	Position	Alleles	Primers	Primer sequences	Annealing T°C	Method
3	57575243	T/C	F	CACGACGTTGTAAAACGACGAAATCGTCACTTGGGAT	52	Pyrosequence
			R	TACTGTCTTAGGATTTTCACAT		
3	57377038	A/G	F	TGACGCTCACAGACTCCCCGCT	55	Pyrosequence
			R	CACGACGTTGTAAAACGACGGACTCAACACTATCCCCTA		
3	57484486	G/T	F	TGATTATCCAAAAACTTGCCTG	55	Pyrosequence
			R	CACGACGTTGTAAAACGACTGCAGAGTCCTGCTGTGA		
3	57457738	T/C	F	AAGAGTAGGGAGTCAAACTG	50	Sanger sequence
			R	CTTTGACAGTCATTGTAAACTC		
3	57420873	C/A	F	TCTTAGGTGCAAATAGTGTA	60	Pyrosequence
			R	CACGACGTTGTAAAACGACCTGAAATTGAGAACCCTGTGAA		
3	57400357	T/C	F	CACGACGTTGTAAAACGACGTATTTGTGGGATGTGTGCTGTGAG	55	Pyrosequence
			R	AGCTGAGCATGGGCACCATTAGGGC		
3	57432981	T/A	F	TAGTTTTTCACCTGGAAAGTTTAGC	55	Pyrosequence
			R	CACGACGTTGTAAAACGACGCAACATAGAACTATCCAAGCTCTA		
3	57441115	A/G	F	TCCAAATTAAAAGAAAATCTCTTCA	55	Pyrosequence
			R	CACGACGTTGTAAAACGACTGGGTAAGATCCTTCAGGTGTATTG		
3	57484658	C/T	F	ATGGCAGGCCTGTTGCTATTTGCAG	55	Pyrosequence
			R	CACGACGTTGTAAAACGACTACCTCCAAGGTCTCTGGATGATGG		
3	57500572	G/A	F	GAACTTACCTCTTTAAATGCTCTC	55	Pyrosequence
			R	CACGACGTTGTAAAACGACTAAATATAGAAAAGAGACAAGATGA		
3	57517383	G/A	F	CACGACGTTGTAAAACGACCAGAGAAACCACAGTGGAGGCCAGA	55	Pyrosequence
			R	GTACAGCAGGGTAAAAACAGTAACA		
8	68708503	C/A	F	GAATATGCATCAAAATGCCTG	55	Pyrosequence
			R	CACGACGTTGTAAAACGACGCTCGCAGACAGTTTAGTTA		
8	68712185	G/A	F	AACGTTCACATCACCATTCACAAC	60	Sanger sequence
			R	GCAACCCTACGTGTGGTCCTTTGAT		
8	68726546	C/T	F	CACGACGTTGTAAAACGACTGATAGGTATTTCACCTCCGAAG	55	Pyrosequence
			R	ATGCGAACATCTTTCTCGGATCTG		
11	67485866	C/T	F	CACGACGTTGTAAAACGACGCAGGAGATTAATAAAGTCAGA	55	Pyrosequence
			R	GAGCATGGGTTTTAAGCAACAG		
11	67538032	G/T	F	GATGGATTGCTAGCAGAATGAA	50	Pyrosequence
			R	CACGACGTTGTAAAACGACGTAGTCGCTAATCACGATCTAT		
11	67516041	A/G	F	CACGACGTTGTAAAACGACCTGCCCTGCGTTCCTCTATCCC	50	Pyrosequence
			R	CTGAAAACTCGCCCGAACCT		
11	67538806	T/C	F	TGACATCAAATCCGACGATGAG	50	Pyrosequence
			R	CACGACGTTGTAAAACGACGATCAGCACCGTCCCGCTTTC		
11	67583604	T/C	F	CTCTGGTAAGTACCAGCCCTTTA	60	Sanger sequence
			R	CACGATATACTAATGTTCGGTGTG		
24	36011545	T/C	F	ATCAGTTGATACCAAGCTTCTCCTA	55	Pyrosequence
			R	CACGACGTTGTAAAACGACATCATTTCAGCAAGTTTAGCAACAC		
24	36075020	T/G	F	GTTGCAGCCAAACGTGCAGCATTTC	55	Pyrosequence
			R	CACGACGTTGTAAAACGACTGCTACAGGGAGGAAAGGAGATTGC		
24	36075761	A/C	F	ATCTAGGAGGTTGGAGGCTCTCTG	60	Sanger sequence
			R	CATCTTTTGAGGCTTAGCCAGTC		

24	36075732	T/C	F	ATCTAGGAGGTTGGAGGCTCTCTG	60	Sanger sequence
			R	CATCTTTTGAGGCTTAGCCAGTC		
24	36063718	G/A	F	CACGACGTTGTAAAACGACGACTACAGTAAACTAGTCG	55	Pyrosequence
			R	GCCTAGGCTGACTTGTCACAGA		
24	36066098	A/G	F	CAACATACCTCCCTTAATGTC	50	Sanger sequence
			R	TGCAGAGTCTGGTGGTGAATGT		
24	36087012	C/G	F	CACGACGTTGTAAAACGACATTGAGAGACTATAGATAAGCA	55	Pyrosequence
			R	TCATCGAAGTGCCCAGAGT		
32	24542001	T/C	F	AGCCACCAAGGCAGATGGAATTAC	55	Pyrosequence
			R	CACGACGTTGTAAAACGACTCTCTTATAGTTCAAACACGG		
32	24556037	G/A	F	AATTCAGCAAGTTACCTTATCA	50	Sanger sequence
			R	CTTAGCATTATACTCTCTTGGT		
32	24606503	T/C	F	TTCCTCAGGTTGAGGGTT	52	Pyrosequence
			R	CACGACGTTGTAAAACGACGCTTCAATGTACTCTTGTAGTT		
32	24667283	C/T	F	GATAAGGGTTGAAAGAATAGGCAAG	50	Sanger sequence
			R	CAAACAGCCTAGAGTCACTTTCT		
32	24667774	C/G	F	GATAAGGGTTGAAAGAATAGGCAAG	50	Sanger sequence
			R	CAAACAGCCTAGAGTCACTTTCT		
32	24672221	G/A	F	TGAGTCTCGAGGATTGAATGACT	50	Sanger sequence
			R	CCTAGGGTGATTTTGTGTAAGCT		
32	24827518	C/T	F	CACGACGTTGTAAAACGACGGTTCAAATCCCAAGATCAAGT	50	Pyrosequence
			R	GGTTCAAATCCCAAGATCAAGT		
32	24890208	A/G	F	CACGACGTTGTAAAACGACAGGTAATGGAGTAATGTAAGT	52	Pyrosequence
			R	GGAAAATTTAGTGGCCTGTGTT		
32	24985562	G/A	F	CTATTTTTGACAATAAAGCATC	50	Pyrosequence
			R	CACGACGTTGTAAAACGACTACAATTAAGGAAACGAATTGC		
32	24987404	G/A	F	CAGTCGCGGTCGCTTCTCATCT	57	Pyrosequence
			R	CACGACGTTGTAAAACGACGCAGCTGCAGAGCTTTATGAC		
32	25007496	A/G	F	TAAAAGCATGAGGGAAACAGCATC	50	Sanger sequence
			R	TAATTCTTTCAGTGAGGGCATTATAG		
32	25305524	A/G	F	CCCCGAGCTACAGAGATGGA	55	Sanger sequence
			R	AGCACAGCCCCTGTGAAAAT		
32	25079168	A/G	F	CACGACGTTGTAAAACGACGCTTTAGAGCAACCACCTAA	55	Pyrosequence
			R	TCCTTGTGTATCCCATGCCAA		
32	25363099	G/A	F	CACGACGTTGTAAAACGACTGCAAAATTCAACTGTAATG	55	Pyrosequence
			R	CCATACATCACCGACCCTCAGC		
32	25485961	A/G	F	CATGGCAACCCAAAGGCAAC	55	Sanger sequence
			R	CCCCTTCACAGATACCCTGC		
32	25512953	T/A	F	GAAAGATTCTAAATCCTTGAAC	50	Pyrosequence
			R	CACGACGTTGTAAAACGACTCTAATAGCATCATTTATCA		
32	25537276	C/T	F	TGGTCTGAGCCTGAAAGTGG	55	Sanger sequence
			R	TGCTGCTGCTGTTAAAGGGT		
32	25537876	A/G	F	TGGTCTGAGCCTGAAAGTGG	55	Sanger sequence
			R	TGCTGCTGCTGTTAAAGGGT		
32	25642357	A/T	F	TCTCTCCTCTTTAGCTTCTGCC	55	Sanger sequence
			R	TCCCTGGTTGGAAATGAGCC		

32	25702963	A/G	F	TGCGCTTTAAGACACGTGGA	55	Sanger sequence
			R	GCAAAGTGCAAGCAAGGTGA		
32	25714903	T/C	F	CACGACGTTGTAAAACGACTGAGGTCGAAGGAGGAGAGATG	50	Pyrosequence
			R	ATCCCTAGCATACTAGACTTTC		
32	25718852	C/T	F	GTTCCAGGGAGCCTGGTGCCTG	57	Sanger sequence
			R	CACGACGTTGTAAAACGACTGAACAGTATTACGACATCTTC		
32	25779083	A/C	F	TCCGTCAAATTGTTTCTCATGTTGA	50	Sanger sequence
			R	TGAGTACCTTAACAGTTCAGAGC		
32	25798353	G/A	F	CACGACGTTGTAAAACGACATTAAGAATAGATCCTCCTACA	55	Pyrosequence
			R	ACTATCTACTGGCAGGTATCCA		
32	26115349	A/T	F	GTCAGCCTCCTGGGTATTTGTA	55	Pyrosequence
			R	CACGACGTTGTAAAACGACTGGAACTGCTGTTTTAATGT		
