# Supplementary material for: Multiple Changes of Gene Expression and Function Reveal Genomic and Phenotypic Complexity in SLE-like Disease
Source: PLoS Genet. 2015 Jun 9;11(6):e1005248. doi: 10.1371/journal.pgen.1005248 (PMC4461293; doi:10.1371/journal.pgen.1005248)
Supplement: S12 Table — (PDF) [file pgen.1005248.s019.pdf]

Table S12. Primers for qRT-PCR.

Gene/primers	Sequence	Annealing T°C	amplicon length
AP3B2			
AP3B2-qPCR-for<br>Rev-AP3B2-qPCR	AGCTGTGTACCCAAACCCGGC<br>TCAGCTTTCCTTGTTCCTTCTT	63	114 bp
WHAMM			
WHAM-ex7-8-for<br>WHAM-Rev-ex9q	AGTATCCAGGTGAAAAGAGACAAG<br>ACCTGGACATCTCTCTTTAAATGC	62	120 bp
HOMER2			
Ex2-Homer2-forw<br>Rev-Homer2-ex3	CTACTTCTACGATGTCACCAGGAATAG<br>AACACCGTGTTGGCTCTGCTGTC	60	153 bp
VRK1			
VRK1-ex10-for<br>Rev-VRK1-ex11	AATATTGCTGGTTTGATGGACAAAT<br>CTTGCCATCATCCTTGCTTCCTAT	63	174 bp
PTPN3			
PTPN3-ex2-3-for<br>Rev-PTPN3-ex4-5	AGTCGACTCTCTGAGATGGCTG<br>CAAATGCCTGGTTTGTTCTTGCT	60	142 bp
WFDC3			
WFDC3-ex3-4-for<br>Rev-ex5-6-WFDC3	GGCATTCCAGAGGGGAGGAAA<br>CACCCCGCCCTCCCTTAAT	66	302 bp
DNTTIP1			
DNTTIP-ex10-11-for<br>Rev-DNTTIP-ex12-13	CCGCACCTCTTTAAGTATGCAG<br>AAGTCCAGGCATCCTCTATAGTCATC	60	158 bp
MTTP<br>MTTP-for-ex15-16<br>Rev-ex17-MTTP	CAGATCATTCTCAGGAGCTTCAGT<br>GTCCACAGTCATGCTGCCAATTA	63	176 bp
DAPP1			
DAPP1-Ex1-2-f<br>Rev-ex3-DAPP1	AGGAATTGGGGTGGTATCACG<br>CAACATGAAAGTGTTTGACAGAGTC	60	165 bp
LAMTOR3			
LAMTOR-ex5-6-for<br>Rev-ex7-LAMTOR3	AACACCTACCAGGTGGTTCAATTCA<br>TGCCAGATTAAGAAACTTCCACCACT	60	157 bp
DNAJB14			
DNAJB14-ex6-7-for<br>Rev-NEW-ex8-DNAJB14	AGATCTGGATCAGGGCAAAC<br>TACTTTGGCTGCATACTGCATATC	60	207 bp

H2AFZ			
H2AFZ-ex3-4-for<br>Rev-ex4-5-H2AFZ	ACCGCAGAGGTACTTGAATTG<br>GTGGAATAACACCACCACCAG	60	151 bp
DDIT4L			
DDIT4L-ex1-for<br>Rev-ex1-2-DDIT4L	CATGGTGGCAACTGGCAGTTTGA<br>CAGTAGTCAAAATCATTTAGCAGGC	60	105 bp
EMCN			
EMCN-For-qPCR<br>Rev-EMCN-qPCR	CAGACCCAGGCACACCAGAA<br>TGCAGAGTGCTCACCAGACTCAT	60	110 bp
PPP3CA			
PPP3CA-ex2-for<br>Rev-ex3-PPP3CA	TAATAACAGAAGGGGCTTCAATTC<br>CTGTCAACATAGTCCCCTAAGAAG	60	175 bp
BANK1			
K9-BANK1-for2<br>BANK1-rev-K9	GTATTCAGAGGTTCTGAGGACTA<br>TCACCAGGATTCTCACATGGAAT	63	176 bp
***TBP* house-keeping gene**			
TBP-ex5-forw<br>Rev-ex6-7-TBP	TCAGTTCTGGGAAGATGGTGTGTA<br>CTCTGGCTCGTAACTGCTAAACT	63	218 bp
